# Longitudinal clustering of health behaviours and their association with multimorbidity in older adults in England: A latent class analysis

**DOI:** 10.1371/journal.pone.0297422

**Published:** 2024-01-25

**Authors:** Alisha Suhag, Thomas L. Webb, John Holmes

**Affiliations:** 1 Healthy Lifespan Institute, School of Health and Related Research, University of Sheffield, Sheffield, United Kingdom; 2 Department of Psychology, University of Sheffield, Sheffield, United Kingdom; 3 School of Health and Related Research, University of Sheffield, Sheffield, United Kingdom; University of Auckland, NEW ZEALAND

## Abstract

**Background:**

Health-risk behaviours such as smoking, unhealthy nutrition, alcohol consumption, and physical inactivity (termed SNAP behaviours) are leading risk factors for multimorbidity and tend to cluster (i.e. occur in specific combinations within distinct subpopulations). However, little is known about how these clusters change with age in older adults, and whether and how cluster membership is associated with multimorbidity.

**Methods:**

Repeated measures latent class analysis using data from Waves 4–8 of the English Longitudinal Study of Ageing (ELSA; n = 4759) identified clusters of respondents with common patterns of SNAP behaviours over time. Disease status (from Wave 9) was used to assess disorders of eight body systems, multimorbidity, and complex multimorbidity. Multinomial and binomial logistic regressions were used to examine how clusters were associated with socio-demographic characteristics and disease status.

**Findings:**

Seven clusters were identified: *Low-risk* (13.4%), *Low-risk yet inactive (*16.8%), *Low-risk yet heavy drinkers* (11.4%), *Abstainer yet inactive* (20.0%), *Poor diet and inactive* (12.9%), *Inactive*, *heavy drinkers* (14.5%), and *High-risk smokers* (10.9%). There was little evidence that these clusters changed with age. People in the clusters characterised by physical inactivity (in combination with other risky behaviours) had lower levels of education and wealth. People in the heavy drinking clusters were predominantly male. Compared to other clusters, people in the *Low-risk* and *Low-risk yet heavy drinkers* had a lower prevalence of all health conditions studied. In contrast, the *Abstainer but inactive* cluster comprised mostly women and had the highest prevalence of multimorbidity, complex multimorbidity, and endocrine disorders. *High-risk smokers* were most likely to have respiratory disorders.

**Conclusions:**

Health-risk behaviours tend to be stable as people age and so ought to be addressed early. We identified seven clusters of older adults with distinct patterns of behaviour, socio-demographic characteristics and multimorbidity prevalence. Intervention developers could use this information to identify high-risk subpopulations and tailor interventions to their behaviour patterns and socio-demographic profiles.

## Introduction

A growing number of older adults are living with multimorbidity, defined as having two or more chronic diseases [1]. In England, for example, 67.8% of people aged 65 and older are expected to have multimorbidity by 2035 [1]. Multimorbidity is more common in recent cohorts and seems to be emerging earlier in the lifecourse, which places a significant strain on healthcare systems [2, 3]. However, chronic diseases (e.g., type-2 diabetes, coronary heart disease, chronic obstructive pulmonary disease) and some cancers that form a large proportion of the multimorbidity burden, have well-established modifiable risk factors, suggesting that there are opportunities for prevention [4]. Among such modifiable risk factors–health risk behaviours such as smoking, poor nutrition, alcohol consumption, and physical inactivity (collectively termed ‘SNAP’ behaviours) account for nearly one-third of disability-adjusted life years from chronic conditions [5]. Most preventative interventions for older adults focus on single behaviours [6]. However, research shows behavioural risk factors typically cluster in specific combinations within distinct populations. This suggests that interventions targeting these clusters may be more appropriate [7]. However, designing such interventions is challenging because individuals’ health behaviours not only cluster but may also change over time [8].

Additionally, engaging in multiple health-risk behaviours can have a negative impact on health that is greater than the sum of their individual effects [9]. While epidemiological studies have attempted to understand the combined impact of multiple behaviours on health, they often rely on simple indices that count the number of co-occurring behaviours without considering which behaviours, or combinations of behaviours, are driving the risk [10, 11]. Other studies have tackled this issue by examining the health risks associated with combinations of behavioural factors in dyads, triads, and tetrads [12]. However, they tend to overlook less common combinations involving multiple risk behaviours because of sparse data [12]. Clustering techniques, such as repeated measures latent class analysis (RMLCA), can better address these issues by grouping individuals with similar patterns of health-risk behaviours over time into clusters [10].

Identifying clusters of health-risk behaviours–and examining whether and how behaviours within these clusters change as people age–can inform interventions by: a) identifying high-risk populations (e.g. subgroups exhibiting combinations of behaviours that entail the greatest risk), b) informing the selection of target behaviours (e.g. those with the greatest health impact or highest reach), and c) detecting potential spillover effects (i.e. where targeting one health behaviour leads to compensatory changes in other behaviours) [13]. For instance, when some individuals reduce cigarette smoking, the reward value and consumption of ’treat foods’ increases, resulting in weight gain [13].

Previous studies that have used clustering techniques to analyse multiple behaviours in older adults have typically been cross-sectional, and so cannot test whether behaviour clusters change over time and if these changes affect long-term health [14–16]. While two studies have used longitudinal clustering to study a subset of SNAP behaviours in older adults [17, 18], none have examined the relationship between these clusters and multimorbidity. In addition, the aforementioned studies limit themselves to a basic definition of multimorbidity (i.e. a simple count of the number of diseases), which overlooks differences between diseases within one system and those spanning multiple systems [19]. This is crucial, as multimorbidity may have a larger impact on overall health if it arises out of chronic conditions in different body systems that are likely to compete for treatment, rather than closely related comorbidities that might have shared pathophysiology or shared approaches to management [20]. The construct of complex multimorbidity, defined as “the co-occurrence of three or more chronic conditions affecting three or more different body systems within one person without an index chronic condition”, addresses these issues by focusing on chronic conditions affecting multiple body systems [21, p1]. This definition also has the advantage of identifying the number and types of specialised health services involved in a patient’s care, thus identifying individuals with more complex needs [21]. Complex multimorbidity might also better reflect the biology of ageing as it involves a simultaneous breakdown or dysfunction of multiple, separate body systems, making it a more reflective measure to study in older adults [22].

Equally crucial is to recognise that multimorbidity is associated with social and economic determinants [23] that can add to (or interact with) behavioural determinants. For example, advanced age, female gender, low socioeconomic status, and education have all been identified as significant risk factors for the onset of multimorbidity [24]. These findings align with the Social Determinants of Health (SDoH) framework, which describes how broader societal structures–from economic policies to social norms–shape and segment populations hierarchically based on gender, race, education, occupation, and income [25, 26]. This stratification then directly and indirectly influences health outcomes. The present research therefore incorporates insights from the SDoH framework to examine whether socio-demographic factors predict membership within risk behaviour clusters. Given that socio-demographic determinants can not only shape individual health behaviours but also predict health outcomes through complicated multifactorial pathways, we will also adjust for them in examining the relationship between health behaviours and outcomes [24].

### The present research

The present research analyzes data from a longitudinal panel of older adults in England to: i) explore how the SNAP behaviours cluster over time in older adults, ii) investigate how membership in different behavioural clusters varies by socio-demographic characteristics, and iii) examine which, if any, behavioural clusters are prospectively associated with multimorbidity over time.

## Methods

### Study design

We analysed secondary data from the English Longitudinal Study of Ageing (ELSA)–a nationally representative, ongoing panel study of community-dwelling adults aged 50 and over at baseline, in England [27]. ELSA collects biennial data on mental and physical health, finances, and attitudes around ageing using computer-assisted interviews and questionnaires [27]. This study followed the STROBE (Strengthening the Reporting of Observational Studies in Epidemiology) guidelines (See Supplementary Section 5 in S1 Appendix) [28]. Ethical approval for ELSA was obtained from the National Research Ethics Service. All participants provided written informed consent. Separate ethical approval and consent were not required for our analyses because data were fully anonymised.

### Sample selection and exclusion criteria

Our analysis used data from 5,429 respondents to the core questionnaire across six waves from Wave 4 (2008–2009) to Wave 9 (2018–2019). We applied the longitudinal weights that were provided with the dataset and had been derived using information spanning from Wave 4 to Wave 9 to reduce drop-out bias. Wave 4 was selected as the baseline because, although data on health behaviours was available from Wave 3, longitudinal weights were only available from Wave 1 or Wave 4. Choosing Wave 3 as the baseline would have resulted in the loss of data on approximately 2000 participants due to longitudinal weighting.

**Fig 1:**
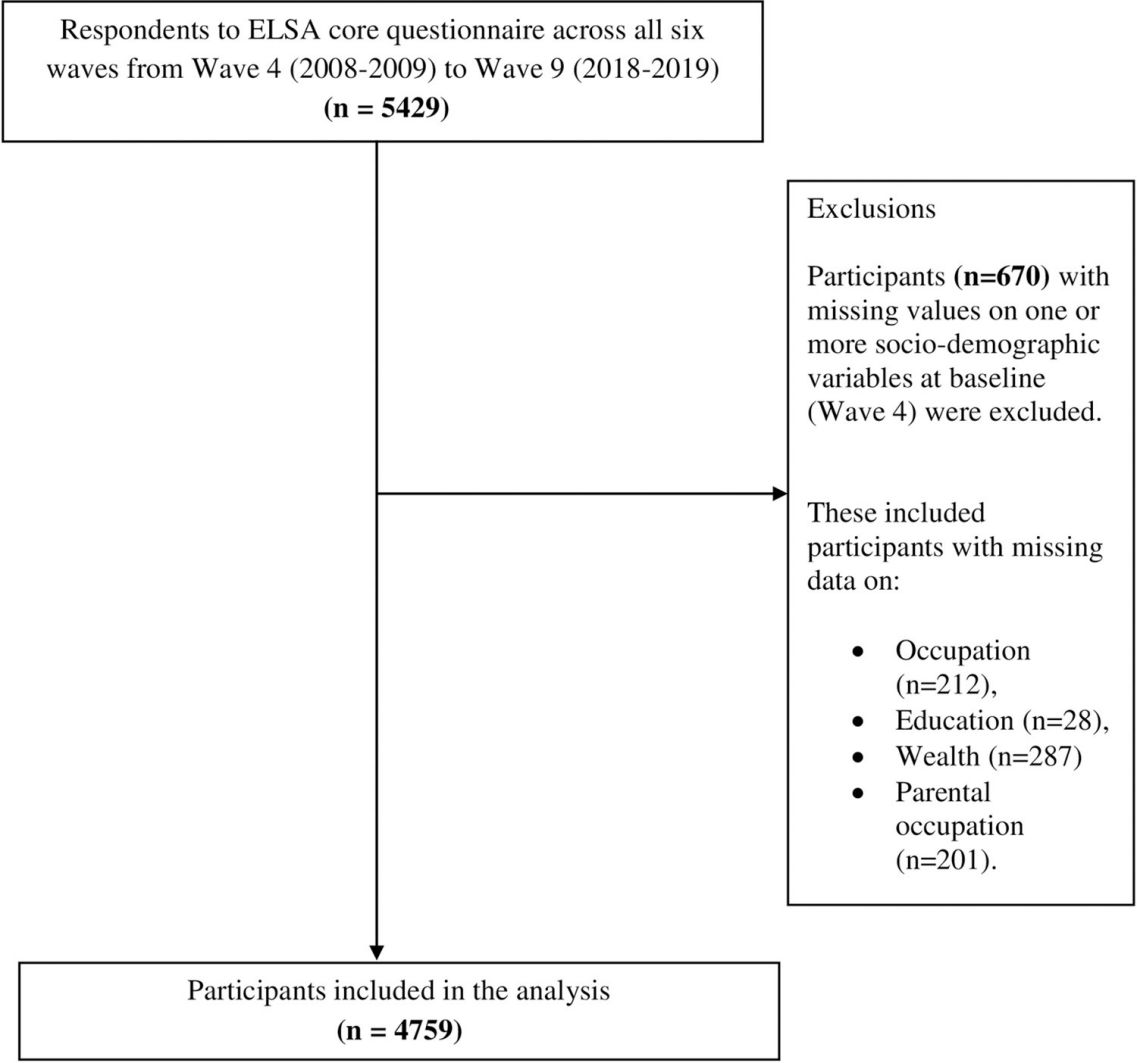
Study flow chart.

Participants (n = 670) with missing values on socio-demographic variables were removed using listwise deletion, leaving a final sample of 4759 participants (87.6% of the original sample; see Fig 1). We chose to use listwise deletion because the MPlus v8.5 software package does not support handling missing data for socio-demographic predictors in a latent class analysis (see Fig 1). More specifically, data was missing for occupation (n = 212), education (n = 28), wealth (n = 287), and parental occupation (n = 201). Given that no socio-demographic variable had more than 5% missing data, a threshold below which multiple imputation is deemed less beneficial, we favoured a complete case analysis [29].

To assess the potential impact of excluding participants with missing data, we compared the included and excluded samples (for details, see Supplementary Section 2 in S1 Appendix). Overall, the absolute differences between the included and excluded samples were not substantial, though differences for some socio-demographic variables (average age, tertiary education, intermediate and professional/managerial occupations) and disease status (complex multimorbidity, respiratory disorders and endocrine disorders) achieved significance due to the relatively large number of participants.

### Measures of health behaviour

Data on health behaviours was taken from Waves 4–8. ELSA provides several measures of SNAP behaviours. Therefore, an online survey was used to gather consensus from experts on how SNAP behaviours should be *defined* for the present analysis (i.e. which measure to choose) and *categorised* into risk groups (i.e. how many categories to divide each behaviour into and what cut-offs to use; see Supplementary Section 1 in S1 Appendix). We describe the agreed measures below.

#### Smoking

We used current smoking status as a measure of smoking. The data comprised participants’ binary (yes/no) response to the question ‘Do you smoke at all nowadays?’

#### Fruit and vegetable intake

In Wave 4, fruit and vegetable intake was assessed with questions such as ‘How much of the following did you eat yesterday?’ for 13 foodstuffs including a ‘small glass of fruit juice’ and ‘salad (cereal bowlfuls)’. However, across Waves 5 to 8, participants were asked ‘how many portions of vegetables–excluding potatoes–do you eat on a given day?’ and ‘how many portions of fruits do you eat on a given day?’ To make fruit and vegetable intake consistent across waves, we used an established method to match Wave 4 and Waves 5 to 8 responses (see Supplementary Fig S1 in S1 Appendix) [30, 31]. Specifically, portions of fruit and vegetable consumed per day were added to create a single variable for each Wave, which was subsequently divided into two categories (<5 or ≥5 portions per day).

#### Alcohol consumption

We included data on alcohol consumption over the last week: Specifically, i) whether the participant consumed alcohol in the last week (yes/no), and ii) the volume of each alcoholic beverage (i.e. beer, wine, and spirits) consumed. Consumption was converted into the number of UK units (1 UK unit = 8g of pure alcohol) using standard assumptions for beverage strength [32]. Consumption was then categorised into four levels based on units consumed per week: *harmful* (>50 units for men, >35 units for women); *hazardous* (>14–50 units for men and >14–35 units for women); *moderate* (14 units or less); *and abstainers* (0 units) [33].

#### Physical activity

Physical activity was recorded in ELSA by asking participants how often they took part in each of three types of physical activity: vigorous-intensity (e.g. running/jogging, swimming, etc.), moderate-intensity (e.g. gardening, cleaning the car, etc.) and low-intensity (e.g. laundry and home repairs). The response categories were: hardly ever/never, one to three times a month, once a week and more than once a week. Following previous research [34, 35], we created a summary index by adding responses to all three questions and classified participants’ levels of physical activity as: *sedentary* (no activity on a weekly basis); *low* (only mild activity at least once a week); *moderate* (moderate but no vigorous activity at least once a week); or *high* (any vigorous activity at least once a week).

### Socio-demographic variables

Socio-demographic variables were taken from Wave 4 and included: *age*, *sex*, *parental occupation*, *own occupation*, *education* and *wealth*. For *parental occupation* and participants’ *own occupation*, the three-class version of the National Statistics—Socioeconomic Classification Scheme [36] was used: professional and managerial occupations, intermediate occupations, and semi-routine and routine occupations. Participants with occupations listed as not classifiable (n = 12) were excluded. *Education* was taken from the Wave 4 IFS derived dataset and grouped into ‘degree/higher’ (National Vocational Qualification NVQ4/NVQ5/degree or equivalent), ‘intermediate’ (higher education below degree, NVQ3/GCE A-level equivalent, NVQ2/GCE O-level equivalent, NVQ1/CSE other grade equivalent or foreign/other), ‘no qualifications’ [37]. *Wealth* was chosen as the most appropriate economic indicator for participants aged 50 and above, as it better reflects their financial resources during active professional life and retirement compared to income. The ELSA derived dataset defines wealth as the net total non-pension wealth including property, possessions, housing, investments, savings, artwork, jewellery, and net of debt reported at the household level (i.e. an individual or a couple living at the same address who make joint financial decisions) [38]. Wealth was grouped into tertiles to reduce measurement error and facilitate comparisons of health measures across equally sized groups within the population. All variables (except age) were converted into dummy variables, with the lowest category serving as the reference group.

### Disease status

We assessed the disease status of 25 physical and mental health conditions recorded at each wave as listed in Table 1, to evaluate basic multimorbidity and complex multimorbidity [22]. Disease data was collected from the baseline Wave 4, and from the final Wave 9. Participants were asked whether they still had the condition diagnosed by a doctor that they had reported previously and, if not, whether they could report a new condition.

**Table 1:** Morbidities used to ascertain multimorbidity and complex multimorbidity.

Body system disorders	Morbidities
**1. Eye disorders**	1. Glaucoma
	2. Macular degeneration
	3. Cataracts
**2. Circulatory disorders**	1. High blood pressure
	2. Angina
	3. Heart Attack
	4.Congestive heart failure
	5. Heart murmur
	6. Abnormal heart rhythm
	7. Stroke
**3. Endocrine, nutritional and metabolic**	1. Diabetic eye disease
	2. Diabetes
**4. Musculoskeletal and connective system**	1. Osteoporosis
	2. Arthritis
**5. Respiratory**	1. Lung disease
	2. Asthma
**6. Neoplasms**	1. Cancers
**7. Nervous disorders**	1. Parkinson’s disease
	2. Alzheimer’s disease
	3. Hallucinations
**8. Mental and behavioural**	1. Anxiety
	2. Depression
	3. Emotional problems
	4. Mood swings
	5 Dementia

*Note*. Adapted from Singer et al. [22]

Researchers have measured multimorbidity using various methods [19]. Given the lack of a uniform approach, we adopted the most widely cited and accepted definition of basic multimorbidity, which identifies it as having two or more chronic conditions [39]. Consequently, we coded respondents as “yes” for multimorbidity if they had two or more conditions from the 25-condition list and as “no” otherwise.

Complex multimorbidity was defined as having three or more conditions affecting three or more body systems [21, 22]. We based our selection of body system disorders (listed in Table 2) for calculating complex multimorbidity on a previous study using the ELSA dataset [22]. This study identified eight body systems as outlined by the International Classification of Diseases 10th Revision system [40]: eye disorders; circulatory disorders; nervous disorders; mental and behavioural problems; neoplasms; respiratory disorders; endocrine, nutritional and metabolic disorders; and musculoskeletal and connective system disorders. Using the self-reported presence or absence of three or more body system disorders, we derived a binary variable representing complex multimorbidity.

**Table 2:** Baseline socio-demographic data of the final sample (n = 4759).

Baseline socio-demographic characteristics	Included sample (n = 4759)
	**Mean (SD)**
Average Age	62.9 (8.1)
	**N (%)**
Male	2081 (43.7)
Female	2678 (56.3)
Parental Occupation–Semi-routine and routine	1299 (27.3)
Parental Occupation–Intermediate	1530 (32.1)
Parental Occupation–Professional/managerial	1930 (40.6)
Occupation–Semi-routine and routine	1646 (34.6)
Occupation–Intermediate	1245 (26.2)
Occupation–Professional/managerial	1868 (39.3)
Education–No qualifications	958 (20.1)
Education–Intermediate	2807 (59.0)
Education–Degree/higher	994 (20.9)
Wealth–First Tertile	1260 (26.5)
Wealth–Second Tertile	1615 (33.9)
Wealth–Third Tertile	1884 (39.6)

### Statistical analysis

RMLCA was used to examine whether there were distinct classes of respondents who had similar patterns of SNAP behaviours over time. RMLCA was chosen as it adopts a probabilistic model-based approach for capturing the number and composition of clusters, handles categorical data well, and allows for reliable interpretation and replication of patterns uncovered in the data. MPlus v8.5 software and R version v4.0.3 [41, 42] was used to conduct the RMLCA.

A two-stage approach was used. In the first stage, the optimal number of classes (i.e. clusters) was determined (Aim 1). For this, data on the four health behaviours across the five waves was entered as independent data points to create a series of LCA models with increasing numbers of latent classes (i.e. clusters) until the model fit stopped improving. The fit of the models was evaluated using several indices: Consistent Akaike’s Information Criterion (CAIC), Bayesian Information Criterion (BIC), adjusted Bayesian Information Criterion (aBIC), Approximate Weight of Evidence Criterion (AWE), Vuong-Lo-Mendell-Rubin Likelihood Ratio Test (VLMR-LRT). More details on the evaluation of model fit are provided in Supplementary Section 3 in S1 Appendix. Missing data on the health behaviours were accounted for by Full Information Maximum Likelihood [42]. To assess the reliability of the class solution, we conducted a split-half replication where the sample was randomly split in half, and the above RMLCA was performed separately on these split samples to see if the solution for the full sample was replicated between these smaller splits. (for details, see Supplementary Section 4 in S1 Appendix). In the second stage, we applied the 3-step method proposed by Bolck, Croon, and Hagenaars [42]. This method assigns individuals to the class that they have the highest posterior probability of belonging to.

To examine the association between socio-demographic characteristics and class membership (Aim 2), we regressed latent classes on socio-demographic variables in a series of multinomial logistic regressions, controlling for the potential inaccuracies in the class assignments, also known as classification errors, that are extracted as part of the 3-step method.

To assess whether the prevalence of each health condition differed across classes (Aim 3), we regressed each health outcome on the latent classes in a series of binomial logistic regressions, adjusting for: i) socio-demographic variables, ii) respective disease at baseline, and iii) classification errors extracted as part of the 3-step method that account for uncertainty in class assignment. Then, for each health outcome, an omnibus Wald chi-square test was conducted to test for differences in disease prevalence across all classes (α = 0.05). If significant, pairwise comparisons were conducted to test for differences in disease prevalence between each pair of classes. The significance level for pairwise comparisons was adjusted using the Bonferroni correction (α = 0.007).

## Results

### Sample characteristics

On average, participants were aged 62.9 years (SD = 8.1) and approximately half were female (56.3%; see Table 2 for baseline demographic data). The sample’s engagement in health behaviours across waves is shown in Table 3. The body system disorders with the highest prevalence were: multimorbidity (57.9%), circulatory disorders (50.3%), and disorders of the musculoskeletal and connective system (47.5%) (see Supplementary Table 3 in S1 Appendix).

**Table 3:** Class-defining indicators (i.e. SNAP behaviours) among participants (n = 4759) included in the latent class analysis.

		Wave 4	Wave 5	Wave 6	Wave 7	Wave 8
		N (%)	N (%)	N (%)	N (%)	N (%)
**Smoking**	Non-smoker	3733 (78.4)	2321 (48.8)	2471 (51.9)	2555 (53.7)	2625 (55.2)
	Smoker	525 (11)	484 (10.2)	432 (9.1)	399 (8.4)	353 (7.4)
	Missing	501 (10.5)	1954 (41.1)	1856 (39)	1805 (37.9)	1781 (37.4)
**Alcohol consumption**	Abstainer	789 (16.6)	929 (19.5)	934 (19.6)	920 (19.3)	961 (20.2)
	Moderate	1780 (37.4)	1763 (37)	1702 (35.8)	1700 (35.7)	1628 (34.2)
	Hazardous	1092 (22.9)	1111 (23.3)	1032 (21.7)	980 (20.6)	966 (20.3)
	Harmful	183 (3.8)	176 (3.7)	177 (3.7)	165 (3.5)	150 (3.2)
	Missing	915 (19.2)	780 (16.4)	914 (19.2)	994 (20.9)	1054 (22.1)
**Physical activity**	Sedentary	22 (0.5)	22 (0.5)	29 (0.6)	27 (0.6)	30 (0.6)
	Low	2345 (49.3)	2389 (50.2)	2466 (51.8)	2578 (54.2)	2658 (55.9)
	Moderate	804 (16.9)	791 (16.6)	762 (16)	767 (16.1)	680 (14.3)
	High	1121 (23.6)	1081 (22.7)	1077 (22.6)	975 (20.5)	914 (19.2)
	Missing	467 (9.8)	476 (10)	425 (8.9)	412 (8.7)	477 (10)
**Fruit and vegetable intake**	< 5 portions/day	1761 (37)	1820 (38.2)	1693 (35.6)	1553 (32.6)	1645 (34.6)
	> = 5 portions/day	2540 (53.4)	2656 (55.8)	2706 (56.9)	2753 (57.8)	2683 (56.4)
	Missing	458 (9.6)	283 (5.9)	360 (7.6)	453 (9.5)	431 (9.1)

### Health behaviour clusters and within-cluster changes

**Fig 2:**
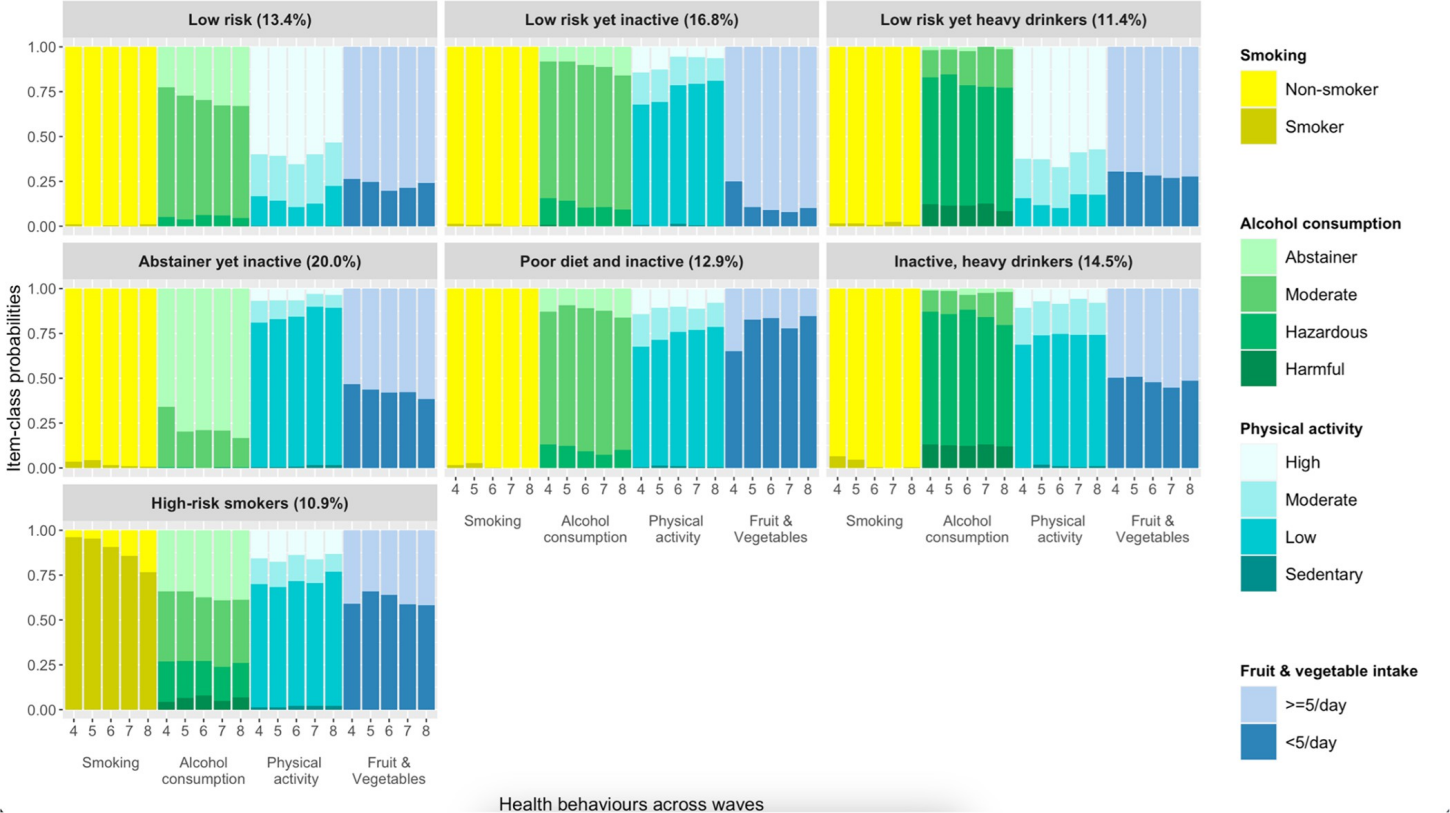
Seven-class model reflecting different clusters of health behaviour across time. *Note*. The x-axis lists each of the four behaviours–smoking, alcohol consumption, physical activity, and fruit and vegetable intake–across five time points. The y-axis provides the average probability for each of the indicators (i.e. four health behaviours) conditional on membership in a given class (i.e. cluster).

After fitting models with one to nine latent classes, a model with seven classes was considered the best fit (model fit statistics are shown in Table 4). The VLMR-LMR test supported a five- and seven-class solution. However, the values of the BIC, SABIC, and CAIC continued to decrease as the number of classes increased, suggesting improved model fit [43]. As the decline in information criteria plateaued around seven classes (screeplot shown in Supplementary Fig S2 in S1 Appendix), we opted for the seven-class model. The entropy and smallest average latent class posterior probability fell within the recommended range (value ≥0.8) for the seven-class model [42]. Furthermore, analysis on the split-half samples also indicated that the 7-class solution was the optimal solution, as identical classes were uncovered in each split-half (full details can be found in Supplementary Section 3 in S1 Appendix). The seven distinct health-risk behaviour classes—hereafter referred to as clusters—were assigned labels. Fig 2 depicts these clusters and the average probability that individuals in each cluster engaged in the four health behaviours—smoking, alcohol consumption, physical activity, and fruit and vegetable intake—over five distinct time points. Most behaviours were fairly stable over time, so unless highlighted below the behaviours that characterise each cluster were similar at each time point.

**Table 4:** Model fit evaluation information for choosing a latent class model.

K	LL	CAIC	BIC	SABIC	AWE	VLMR LRT p-value	Entropy	Smallest average latent class posterior probability	Smallest class size (%)
1	-64393.26	128866.51	129125.23	128998.12	128796.5	-	-	1	100
2	-60051.70	120265.39	120789.28	120531.90	120123.3	<0.001	0.810	0.943	42.5
3	-56767.60	113779.20	114568.27	114180.60	113565.1	<0.001	0.870	0.936	12.4
4	-55061.43	110448.86	111503.11	110985.15	110162.7	<0.001	0.860	0.892	11.6
5	-54186.53	108781.06	110100.49	109452.25	108422.9	<0.001	0.830	0.823	11.2
6	-53544.39	107578.79	109163.40	108384.88	107148.6	0.770	0.830	0.819	11
**7**	**-52947.80**	**106467.60**	**108317.39**	**107408.58**	**105965.4**	**<0.001**	**0.820**	0.820	10.9
8	-52464.47	105582.94	107697.91	106658.82	105008.7	0.760	0.820	0.819	3
9	-52081.57	104899.14	107279.29	106109.92	104252.9	0.760	0.820	0.810	3.2

*Note*: *n* = 4759; K = number of classes (the nine-class model failed to converge); LL = model log likelihood; BIC = Bayesian information criterion; SABIC = sample size adjusted BIC; CAIC = consistent Akaike information criterion; AWE = approximate weight of evidence criterion; VLMR-LRT = Vuong-Lo-Mendell-Rubin adjusted likelihood ratio test; p-value significance <0.05.

Participants in the *Low-risk* cluster (13.4% of the sample) maintained high levels of physical activity and adequate (≥5 per day) fruit and vegetable intake across time. They also had a consistently high probability of drinking at moderate levels, although the proportion of people who abstain from alcohol rose across waves.

Participants in the *Low-risk yet inactive* cluster (16.8%) displayed low levels of physical activity. However, they consumed the recommended portions of fruit and vegetables, and had moderate alcohol intake.

Participants in the *Low-risk yet heavy drinkers* cluster (11.4%) exhibited patterns of smoking, physical activity, and diet similar to those in the *Low-risk* cluster. However, participants in the *Low-risk yet heavy drinkers* cluster were more likely to drink at hazardous and harmful levels.

The largest proportion of participants fell in the *Abstainer yet inactive* cluster (20%). Although participants in this cluster displayed low levels of physical activity, they were also the least likely to consume alcohol at harmful or hazardous levels and had the highest probability of abstaining from alcohol which steadily rose across waves.

Participants in the *Poor diet and inactive* cluster (12.9%) had a similar profile to the *Low-risk yet inactive* cluster, except they had a consistently low probability of adequate fruit and vegetable intake—the lowest of any cluster.

Participants in the *Inactive*, *heavy drinkers* cluster (14.5%) had a high probability of drinking at hazardous and harmful levels while displaying consistently low levels of physical activity.

Finally, participants characterised as *High-risk smokers* cluster (10.9%) had a high probability of smoking, albeit declining over time, low levels of physical activity, and inadequate fruit and vegetable intake.

### Clusters and socio-demographic characteristics

Table 5 displays the socio-demographic composition of participants in each of the identified clusters and the results from the adjusted multinomial logistic regressions that examined associations between each socio-demographic characteristic and cluster membership.

**Table 5:** Demographics and odds ratios from multinomial logistic regressions examining the association between socio-demographic predictors and cluster membership.

Socio-demographic characteristics	Low risk (n = 13.4%)		Low risk yet inactive (n = 16.8%)		Low risk yet heavy drinkers (n = 11.4%)		Abstainers but inactive (n = 20%)		Poor diet and inactive (n = 12.9%)		Inactive, heavy drinkers (n = 14.5%)		High-risk smokers (n = 10.9%)	
	**(Ref. class)**													
				OR [95% C.I.]		OR [95% C.I.]		OR [95% C.I.]		OR [95% C.I.]		OR [95% C.I.]		OR [95% C.I.]
**Age** **(s.d.)**	61.42 (8.4)	Ref.	65.30 (12)	**1.06 [1.04, 1.08]**	60.31 (7.7)	0.97 [0.96, 1.00]	66.70 (13.2)	**1.07 [1.05, 1.09]**	65.00 (13.5)	**1.06 [1.03, 1.08]**	62.97 (11.3)	**1.03 [1.01, 1.05]**	60.52 (8.7)	**0.97 [0.95, 0.99]**
**Sex**														
Male	45.6%	Ref	35.5%	Ref	67.5%	Ref	25.4%	Ref	51.6%	Ref	69.1%	Ref	45.2%	Ref
Female	54.4%	Ref	64.5%	**1.49 [1.10, 2.02]**	32.5%	**0.40 [0.29, 0.55]**	74.6%	**2.31 [1.68, 3.17]**	48.4%	0.77 [0.55, 1.06]	30.9%	**0.37 [0.27, 0.49]**	54.8%	1.02 [0.75, 1.40]
**Education Level**														
No qualifications	15.5%	Ref	23.4%	Ref	11.3%	Ref	43.9%	Ref	30.1%	Ref	13.4%	Ref	40.5%	Ref
Intermediate	58.1%	Ref	61.4%	0.89 [0.57, 1.39]	52.9%	0.90 [0.53, 1.53]	50.6%	**0.56 [0.38, 0.83]**	60.9%	0.76 [0.49, 1.18]	62.7%	1.24 [0.78, 1.96]	51.2%	**0.44 [0.29, 0.66]**
Degree or higher	26.4%	Ref	15.2%	**0.52 [0.30, 0.88]**	35.8%	0.91 [0.51, 1.63]	5.5%	**0.23 [0.13, 0.40]**	9.0%	**0.32 [0.18, 0.60]**	23.9%	0.84 [0.50, 1.42]	8.3%	**0.21 [0.12, 0.36]**
**Wealth**														
First tertile	15.8%	Ref	25.0%	Ref	9.5%	Ref	47.8%	Ref	37.2%	Ref	20.6%	Ref	50.9%	Ref
Second tertile	35.5%	Ref	37.2%	0.67 [0.43, 1.03]	27.9%	1.17 [0.67, 2.06]	33.9%	**0.38 [0.26, 0.57]**	41.2%	**0.53 [0.34, 0.81]**	30.3%	**0.63 [0.40, 0.97]**	30.5%	**0.33 [0.22, 0.49]**
Third tertile	48.7%	Ref	37.8%	**0.48 [0.31, 0.75]**	62.6%	1.71 [0.99, 2.94]	18.3%	**0.18 [0.12, 0.28]**	21.6%	**0.22 [0.14, 0.36]**	49.1%	0.71 [0.47, 1.09]	18.6%	**0.18 [0.11, 0.28]**
**Occupation—Self**														
Routine/manual	33.3%	Ref	36.8%	Ref	18.5%	Ref	55.8%	Ref	45.8%	Ref	31.4%	Ref	54.1%	Ref
Intermediate	27.0%	Ref	27.7%	1.11 [0.75, 1.64]	26.3%	**1.70 [1.07, 2.71]**	22.6%	0.84 [0.57, 1.22]	28.2%	1.17 [0.77, 1.76]	22.5%	1.03 [0.69, 1.52]	21.9%	0.87 [0.58, 1.30]
Professional/managerial	39.7%	Ref	35.5%	1.32 [0.90, 1.94]	55.2%	**1.95 [1.26, 3.04]**	21.6%	1.02 [0.70, 1.49]	26.0%	1.06 [0.70, 1.62]	46.1%	1.33 [0.93, 1.91]	24.0%	0.96 [0.65, 1.43]
**Parental Occupation**														
Routine/manual	24.2%	Ref	27.3%	Ref	20.8%	Ref	37.5%	Ref	29.5%	Ref	25.1%	Ref	35.9%	Ref
Intermediate	35.0%	Ref	28.8%	0. 78 [0.53, 1.15]	29.7%	0.82 [0.53, 1.25]	34.1%	0.79 [0.54, 1.14]	38.5%	1.06 [0.71, 1.60]	28.9%	0.77 [0.52, 1.13]	40.2%	0.96 [0.66, 1.40]
Professional/managerial	40.8%	Ref	43.9%	1.14 [0.78, 1.67]	49.5%	1.11 [0.73, 1.67]	28.4%	0.85 [0.58, 1.23]	32.0%	1.10 [0.71, 1.71]	46.0%	1.18 [0.81, 1.71]	23.9%	0.76 [0.51, 1.14]

*Note*. Odds Ratios [95% Confidence interval] are from BCH multinomial logistic regression analysis; Ref = Reference cluster. **Bold values** are statistically significant at the significance level (p = 0.05). All clusters are compared to the Reference cluster—*Low-risk*. Each odds ratio is adjusted for the remaining socio-demographic variables in the model.

Compared to participants in the *Low-risk* cluster (which served as the reference group), participants characterised as *Low risk yet inactive*, *Abstainers but inactive*, *Poor diet and inactive*, and *Inactive*, *heavy drinkers* were more likely to be older. In contrast, individuals in the *High-risk smokers* cluster were more likely to be younger than participants in the *Low-risk* cluster. However, while these age-related associations are notable, they were weaker than the associations found with other socio-demographic factors.

Men made up around 70% of two clusters: *Low-risk yet heavy drinkers* and *Inactive*, *heavy drinkers*. By contrast, the two clusters labelled *Low-risk yet inactive* and *Abstainers but inactive* were predominantly female, with the likelihood of female membership in these groups being 1.5 to 2 times higher than the *Low-risk* cluster (reference group).

Notably, the *Low-risk yet inactive* and *Abstainers but inactive* clusters were less likely to comprise individuals with degree-level education or those belonging to higher wealth tertiles, compared to the *Low-risk* cluster. Similarly, participants characterised as *High-risk smokers* and *Poor diet and inactive* were also less likely to belong to the higher tiers of both wealth and education compared to participants in the *Low-risk* cluster.

Individuals characterised as *Low-risk yet heavy drinkers* were more likely to have professional or managerial occupations than those in the *Low-risk* cluster.

### Health behaviour clusters and disease status

The prevalence of multimorbidity, complex multimorbidity, respiratory disorders, and endocrine, nutritional, and metabolic disorders differed significantly across clusters (omnibus Wald test χ^2^(df = 6) multimorbidity = 14.954, p = 0.021; χ^2^(df) = 6 complex multimorbidity = 31.326, p <0.001; χ^2^(df = 6) respiratory disorders = 35.998, p <0.001; and χ^2^(df = 6) endocrine, nutritional and metabolic disorders = 53.201, p <0.001, respectively). To further investigate which clusters differed in the proportions of participants with each disease profile, we conducted pairwise comparisons between clusters for respiratory diseases, multimorbidity, complex multimorbidity, and endocrine, nutritional, and metabolic disorders, as shown in Table 6. Results were adjusted for disease status and socio-demographic variables at baseline Wave 4 (see Table 6; for unadjusted results, see Supplementary Table S5 in S1 Appendix).

**Table 6:** Pairwise comparisons of disease status prevalence across clusters (adjusted for i) the specific disease at baseline Wave 4, and ii) socio-demographic variables–sex, age, parental occupation, own occupation, education level and wealth).

Health conditions			1	2	3	4	5	6	7
			Low risk (Ref. class)	Low risk yet inactive	Low risk yet heavy drinkers	Abstainers but inactive	Poor diet and inactive	Inactive, heavy drinkers	High-risk smokers
			(n = 13.4%)	(n = 16.8%)	(n = 11.4%)	(n = 20%)	(n = 12.9%)	(n = 14.5%)	(n = 10.9%)
Multimorbidity	1	0.469		-0.179	0.015	-0.242	-0.128	-0.125	-0.108
** **				**(0.007)**	(0.279)	**(0.003)**	(0.300)	**(0.005)**	(0.096)
	2	0.648	**0.179**		0.194	-0.063	0.051	0.054	0.071
			**(0.007)**		(0.094)	(0.760)	(0.115)	(0.983)	(0.320)
	3	0.454	-0.015	-0.194		-0.257	-0.143	-0.14	-0.123
			(0.279)	(0.094)		(0.053)	(0.999)	(0.114)	(0.549)
	4	0.711	**0.242**	0.063	0.257		0.114	0.117	0.134
			**(0.003)**	(0.760)	(0.053)		(0.073)	(0.745)	(0.202)
	5	0.597	0.128	-0.051	0.143	-0.114		0.003	0.02
			(0.300)	(0.115)	(0.999)	(0.073)		(0.130)	(0.560)
	6	0.594	**0.125**	-0.054	0.14	-0.117	-0.003		0.017
			**(0.005)**	(0.983)	(0.114)	(0.745)	(0.130)		(0.344)
	7	0.577	0.108	-0.071	0.123	-0.134	-0.02	-0.017	
** **			(0.096)	(0.320)	(0.549)	(0.202)	(0.560)	(0.344)	
**Complex Multimorbidity**	1	0.18		**-0.148**	0.057	**-0.226**	-0.09	-0.085	**-0.109**
				**(0.007)**	(0.287)	**(<0.001)**	(0.352)	(0.009)	**(0.006)**
	2	0.328	**0.148**		**0.205**	-0.078	0.058	0.063	0.039
			**(0.007)**		**(<0.001)**	(0.350)	(0.100)	(0.836)	(0.869)
	3	0.123	-0.057	**-0.205**		**-0.283**	-0.147	**-0.142**	**-0.166**
			(0.287)	**(<0.001)**		**(<0.001)**	(0.057)	**(0.001)**	**(<0.001)**
	4	0.406	**0.226**	0.078	**0.283**		0.136	0.141	0.117
			**(<0.001)**	(0.350)	**(<0.001)**		(0.014)	(0.263)	(0.471)
	5	0.27	0.09	-0.058	0.147	-0.136		0.005	-0.019
			(0.352)	(0.100)	(0.057)	(0.014)		(0.158)	(0.087)
	6	0.265	0.085	-0.063	**0.142**	-0.141	-0.005		-0.024
			(0.009)	(0.836)	**(0.001)**	(0.263)	(0.158)		(0.727)
	7	0.289	**0.109**	-0.039	**0.166**	-0.117	0.019	0.024	
** **			**(0.006)**	(0.869)	**(<0.001)**	(0.471)	(0.087)	(0.727)	
**Respiratory disorders**	1	0.096		-0.051	-0.003	-0.082	-0.018	-0.032	**-0.133**
** **				(0.096)	(0.415)	(0.016)	(0.620)	(0.234)	**(<0.001)**
	2	0.147	0.051		0.048	-0.031	0.033	0.019	**-0.082**
			(0.096)		(0.451)	(0.381)	(0.271)	(0.636)	**(<0.001)**
	3	0.099	0.003	-0.048		-0.079	-0.015	-0.029	**-0.13**
			(0.415)	(0.451)		(0.137)	(0.752)	(0.789)	**(<0.001)**
	4	0.178	0.082	0.031	0.079		0.064	0.05	**-0.051**
			(0.016)	(0.381)	(0.137)		(0.069)	(0.219)	**(0.003)**
	5	0.114	0.018	-0.033	0.015	-0.064		-0.014	**-0.115**
			(0.620)	(0.271)	(0.752)	(0.069)		(0.555)	**(<0.001)**
	6	0.128	0.032	-0.019	0.029	-0.05	0.014		**-0.101**
			0.234	0.636	0.789	0.219	0.555		**(<0.001)**
	7	0.229	**0.133**	**0.082**	**0.13**	**0.051**	**0.115**	**0.101**	
** **			**(<0.001)**	**(<0.001)**	**(<0.001)**	**(0.003)**	**(<0.001)**	**(<0.001)**	
**Endocrine,**	1	0.083		**-0.08**	0.03	**-0.153**	-0.061	-0.016	-0.058
**nutritional and**				**(0.005)**	(0.129)	**(<0.001)**	(0.294)	(0.633)	(0.104)
**metabolic**	2	0.163	**0.08**		**0.11**	-0.073	0.019	**0.064**	0.022
**disorders**			**(0.005)**		**(<0.001)**	(0.140)	(0.106)	**(<0.001)**	(0.188)
	3	0.053	-0.03	**-0.11**		**-0.183**	-0.091	-0.046	**-0.088**
			(0.129)	**(<0.001)**		**(<0.001)**	(0.012)	(0.218)	**(0.002)**
	4	0.236	**0.153**	0.073	**0.183**		**0.092**	**0.137**	**0.095**
			**(<0.001)**	(0.140)	**(<0.001)**		**(0.006)**	**(<0.001)**	**(0.006)**
	5	0.144	0.061	-0.019	0.091	**-0.092**		0.045	0.003
			(0.294)	(0.106)	(0.012)	**(0.006)**		(0.108)	(0.644)
	6	0.099	0.016	**-0.064**	0.046	**-0.137**	-0.045		-0.042
			(0.633)	**(<0.001)**	(0.218)	**(<0.001)**	(0.108)		(0.019)
	7	0.141	0.058	-0.022	**0.088**	**-0.095**	-0.003	0.042	
** **			(0.104)	(0.188)	**(0.002)**	**(0.006)**	(0.644)	(0.019)	

*Note*. The estimates are the absolute differences in proportions of participants having the disease in the cluster (in row) minus the cluster (in column). P-values are shown in brackets. **Bold values** are statistically significant at the Bonferroni-corrected significance level (p = 0.007) and indicate the two-tailed p-values for pairwise Wald test for differences in disease proportion for the cluster (in row) minus the cluster (in column).

Participants characterised as *Low-risk* had a lower prevalence of multimorbidity compared to participants in the *Low risk yet inactive*, *Abstainers but inactive*, and *Inactive*, *heavy drinkers* clusters. Participants in the *Low-risk* cluster also had a lower prevalence of complex multimorbidity and endocrine disorders than participants in the *Low-risk yet inactive* and *Abstainers but inactive* clusters. Similarly, the *Low-risk yet heavy drinkers* had a lower prevalence of complex multimorbidity than all other clusters except two, namely, *Low-risk* and *Poor diet and inactive* clusters.

By contrast, *High-risk smokers* had a higher prevalence of respiratory disorders than all other clusters. Individuals in this cluster also a higher prevalence of complex multimorbidity compared to the *Low-risk* and *Low-risk yet heavy drinkers* clusters.

With regards to *endocrine*, *nutritional*, *and metabolic disorders*, participants in the *Low-risk yet inactive* cluster had a higher prevalence than the two clusters characterised by heavy drinking. However, the *Abstainers but inactive* cluster had a higher prevalence of endocrine disorders than all other clusters, except the *Low-risk yet inactive* cluster. *High-risk smokers* also had a higher prevalence of endocrine disorders than the *Low-risk yet heavy drinkers*.

Finally, the *Inactive*, *heavy drinkers* had a higher prevalence of multimorbidity than the *Low-risk* cluster and a higher prevalence of complex multimorbidity than the *Low-risk yet heavy drinkers*.

## Discussion

The present research investigated the relationship between clusters of health-risk behaviours over time and multimorbidity in older adults. We identified seven distinct clusters of behaviour that resemble those found in previous studies from Germany [14], Australia [15], and Taiwan [17] including a cluster characterised by an overall low level of risk, a cluster characterised by physical inactivity, and a cluster characterised by heavy alcohol consumption, non-smoking and low physical activity. Similarly, with the exception of a study focusing on Taiwanese men [17], where the smokers were split across two clusters because of a relatively high prevalence of smoking, the smallest subgroup in each study comprised smokers who exhibited two or more risky behaviours, which parallels our finding that the *High risk smokers* represented the smallest cluster (~11% of the sample). However, our clusters diverged from the findings of a study focusing on six international ageing cohorts, likely because their study: excluded dietary data, included social activity as a behaviour, and used different measures for physical activity, alcohol consumption, and smoking [16].

The present research moved beyond existing research, however, by using longitudinal data to not only examine whether distinct clusters of health behaviours are found in older adults but also whether and how patterns of behaviour within each cluster change over time. We found that patterns of behaviour within the clusters were largely stable over time, with two exceptions: The proportion of current smokers steadily declined in the *High-risk smokers* cluster, while the proportion of alcohol abstainers gradually increased in clusters characterised by moderate or no alcohol consumption (i.e. the clusters labelled *Low-risk* and *Abstainer yet inactive*). Notwithstanding these exceptions, our findings support the idea that SNAP behaviours in older people are fairly stable and likely reflect lifelong habits [8], emphasising the importance of addressing risk behaviours early in the life course to prevent negative health outcomes [44]. Additionally, the finding that behavioural patterns are relatively stable over time suggests that clustering in older adults can be accurately captured by cross-sectional studies.

The clusters also had different socio-demographic profiles. Consistent with alcohol consumption patterns in the UK [32], the two clusters of heavy drinkers were predominantly male. The clusters characterised by physical inactivity but no other risky behaviours (i.e. the *Low-risk yet inactive* and *Abstainer yet inactive* clusters) were primarily female, similar to findings in previous studies [14–17]. *High-risk smokers* were younger on average and, in contrast to previous research, we did not find evidence that high-risk smokers more likely to be men [45]. This may be due to survivorship bias, as smoking is the leading cause of lung cancer deaths, but lung cancer occurs less frequently and has a better prognosis in women [45]. We also found a marked consistency with previous studies looking at clusters of health behaviour among older adults [14, 15, 17], in that we found that clusters characterised by physical inactivity (in combination with other risky behaviours) were less likely to be wealthy or well-educated, suggesting a link between socio-demographic inequalities and health behaviour clustering.

Importantly, identified clusters also differed in their disease status. Participants characterised as *Abstainers but inactive* and *Low-risk yet inactive* had a higher prevalence of complex multimorbidity and endocrine disorders than other low-risk clusters that engaged in health-promoting behaviours (i.e. *Low-risk* and *Low-risk yet heavy drinkers*), and they also had higher rates of multimorbidity compared to the *Low-risk* cluster. Notably, participants in the cluster characterised as *Abstainers but inactive* had a higher prevalence of endocrine disorders than participants in all clusters except *Low-risk yet inactive*. That the cluster characterised by physical inactivity (but no other risk behaviours) was associated with worse health outcomes than clusters characterised by multiple risk behaviours suggests that the relationship between behaviour and health outcomes is more complex than a linear dose–response relationship [46]. Indeed, it is important to recognise the possibility of a bidirectional relationship between physical activity and multimorbidity, since not only is physical activity a risk factor for multimorbidity, but multimorbidity, in turn, can reduce function and reduce adherence to recommended levels of physical activity [47].

Some associations were more straightforward and predictable. For example, we found that *High-risk smokers* had higher rates of respiratory disorders than every other cluster as might be expected. However, *High-risk smokers* also had a higher prevalence of complex multimorbidity and endocrine, nutritional and metabolic disorders compared to the *Low-risk yet heavy drinkers* cluster, a finding that is harder to explain using health behaviour patterns alone. Similarly hard to explain is the finding that *Inactive*, *heavy drinkers* had a higher prevalence of complex multimorbidity than *Low-risk yet heavy drinkers*. One explanation for the lower prevalence of complex multimorbidity and endocrine, nutritional and metabolic disorders could be that, compared to other clusters, the *Low-risk yet heavy drinkers* cluster had the largest proportion of individuals in the highest wealth tertile and in intermediate and professional jobs–indicators of elevated socioeconomic status. This higher socioeconomic status, a known protective factor, may influence health outcomes, as it has consistently been identified as an important determinant of multimorbidity [23]. Thus, examining the interaction between health behaviour clusters and socio-demographic variables on multimorbidity, could further help clarify the patterns of risk. Additionally, our focus on the adverse health effects of risky behaviours might have overshadowed the protective effects of engaging in some behaviours (i.e., adequate fruit and vegetable intake and being physically active) [46]. Recognizing the potential benefits of these behaviours and their associated factors is crucial, as they offer functional, social, and psychological resilience against multimorbidity [48].

### Strengths and limitations

Several strengths distinguish this study. It is the first to examine the association between longitudinal clusters of multiple health-risk behaviours and multimorbidity in older adults. Health behaviour experts helped to choose the most viable of the measures available in ELSA and how these might be used. It uses a robust, model-based, probabilistic approach (namely, RMLCA), demonstrates stable results in split-half replication, is reproducible (i.e. diagnostic criteria and programming codes are accessible) [49]. Furthermore, the results adjust for baseline disease and a range of socio-demographic variables that may confound the relationship between health behaviour and outcomes.

Despite these strengths, the study has some limitations. ELSA relies on self-report data, which can be subject to recall limitations and social desirability bias. Having said this, longitudinal analyses are less susceptible to misclassification bias due to consistent measures across survey waves. It is also important to note that alcohol consumption was only measured for the past week, which may misestimate drinking behaviour for those with inconsistent drinking patterns. Relatedly, missing data are unavoidable in general population cohorts such as ELSA and we had to exclude participants with missing sociodemographic data at baseline. As a result, participants who were included were slightly older, better educated, and more likely to have more intermediate and professional level jobs than those who were excluded. This may limit the generalisability of our findings. Finally, as there are relatively few ethnic minority participants in ELSA, the findings may not generalise to non-white populations.

### Implications

The present research offers new insights into the relationship between clusters of health behaviours and multimorbidity in older adults and has practical implications for interventions to improve health outcomes. For instance, by identifying distinct profiles of risk behaviour, our findings can help to identify high-risk subgroups and select behaviour(s) to target with interventions. For example, our data suggest that targeting physical inactivity, which characterised all five clusters associated with negative health outcomes and represented the majority of the population (70%), could have the greatest potential reach and health impact.

The present findings also demonstrate how targeting different clusters may require tailored approaches. For instance, interventions targeting participants in the *Abstainer yet inactive* cluster, which comprised mostly women and had lower levels of education and wealth, may need to address barriers to physical activity that are specific to their socio-demographic profile. This aligns with existing evidence indicating that interventions tailored to specific target audiences are more effective in promoting changes in multiple behaviours in the general adult population [50, 51] and in patients with chronic conditions [52], than interventions that are not tailored.

## Conclusions

The present research identified seven clusters of older adults with distinct patterns of behaviour that were associated with socio-demographic characteristics and the prevalence of multimorbidity. Notably, we found that the number or combination of risk behaviours alone could not explain why some clusters had worse health outcomes than others. A closer examination of how behaviour clusters interact with socio-demographic characteristics could offer a more nuanced understanding of their combined effect on health outcomes. Integrating this additional layer of complexity into our current study would have made its breadth unmanageable, but it remains an important area for future investigations to explore. Additionally, our findings show that health-risk behaviours tend to be stable as people age, emphasising the importance of addressing them early. Future research should take a lifespan approach to investigate how risk behaviours cluster at earlier life stages.

## Supporting information

S1 Appendix (PDF)
