# Supplementary material for: Longitudinal clustering of health behaviours and their association with multimorbidity in older adults in England: A latent class analysis
Source: PLoS One. 2024 Jan 25;19(1):e0297422. doi: 10.1371/journal.pone.0297422 (PMC10810435; doi:10.1371/journal.pone.0297422)
Supplement: S1 Appendix — (PDF) [file pone.0297422.s001.pdf]

Supplementary Appendix

Table of contents

Section 1: Survey on health behaviour measures	2
Table S1	2-4
Figure S1	5
Section 2: Supplementary Missing Data Analysis	6
Table S2	7
Section 3: Supplementary Statistical Analysis	8
Table S3	9
Figure S2	10
Section 4: Split-half Replication	11
Figure S3	12
Figure S4	13
Figure S5	14
Figure S6	15
Table S5	16-20
Section 5: STROBE Statement	21-27
References	28

Section 1: Survey on health behaviour measures

For the survey, researchers who had published at least one relevant article, as the first author, in the last three years on any of the four SNAP behaviours were identified through the research team's personal networks. Out of an opportunistic sample of 20 researchers, 15 experts participated in our survey. The results of the survey indicated a consensus (defined as agreement of 70% or greater) on the most appropriate measure of fruit and vegetable intake, smoking, and physical activity behaviours (for details, see Supplementary Table 1). Although the results did not indicate a clear consensus for alcohol consumption, the choice with the maximum respondent support was discussed between the authors and chosen.

Table S1. Results of the expert survey on health behaviour measures

SMOKING

1. a) Based on the following measures, which do you think would be the most appropriate measure of smoking for use in our study on behavioural clustering?

Ever smoking status (whether or not the person has ever smoked)	0%
Current smoking status (whether or not the person smokes nowadays)	73%
Number of cigarettes smoked per day (roll-ups can be converted to cigarettes)	27%

1. b) Please explain your answer

- The percentage of current smokers is relatively low, which means that to subdivide smokers into amount smoked would further reduce the statistical power to reliably identify clusters associated with smoking.
- If I can only choose one of the above measures, I think current status is most important to look at clustering of behaviour
- Ever smoking could reflect smoking behaviour from decades previous. Number of cigarettes is important, but to me less important than current status. We know that any amount of smoking is harmful, so it matters less that someone is smoking 10 vs 20 and more that they are smoking at all. I also wouldn't know how to advise on what a numerical cut off should be
- Difference between 0 and some smoking is qualitatively different, and won't be captured by looking at CPD among everyone. Current smoking much more important to current clustering than previous smoking (especially a weak measure of ever rather than ever regular - many have smoked a small number without doing so regularly).
- Most of the literature/policy tends to focus on this binary outcome
- Given the dwindling number of smokers in the population, I think that this is all the information that you need. Cigs per day is a good marker of dependence severity though
- Tricky one to start with and largely depends on the research question! I would say current smoking status, closely followed by ever smoked. Easiest for respondents to answer consistently/accurately and most straightforward to interpret

1. c) How would you categorise the number of cigarettes smoked? Into how many categories would you divide the number of cigarettes smoked?

- I would avoid categorising the numbers and would use the number reported as a continuous variable.
- I might suggest having a category for non-smoker (0 cigs) and then basing the next categories 1-5, 6-10, 10-20 and 20 plus
- I think it's best to ask for a number - it is really hard to work with categories in a meaningful way. It should include the option to say none if they don't smoke at all and a figure less than 1 if they do not smoke every day

ALCOHOL CONSUMPTION

The English Longitudinal Study of Ageing collects the following information on alcohol consumption : - Frequency of alcohol consumption over the past 12 months (Almost every day, 5-6 days/week, 3-4 days/week, 1-2 days/week, once or twice a month, once every couple of months, once or twice a year, not at all in the last 12 months) - Frequency of drinking in the past seven days (number of days) - Quantity of beer (pints), wine (glasses) and spirits (ml) consumed over the past seven days.

2. a) Based on the above stated measures, which do you think would be the most appropriate measure for alcohol consumption for use in our clustering analysis? You may even choose a combination of these e.g., a quantity- frequency measure.

Frequency of alcohol consumption over the past 12 months	15%
Frequency of drinking in the past seven days	15%
Quantity of beer (pints), wine (glasses) and spirits (ml) consumed over the past seven days, converted into UK units	38%
None of these (please indicate your answer in the space below)	31%

If you answered 'none of these', please indicate your answer below.

- Both frequency and quantity (it would not allow me to select both). If there is time, I'd suggest breaking the week down into the weekend and week days. If further time is available I'd suggest asking questions about each day to prompt recall.
- I would also ask if their alcohol consumption had changed in the last 7 days compared to their usual alcohol consumption (or is it similar). Quantity-frequency measure - how many units and how often (days per week)
- I would use a quantity-frequency measure averaged over the previous 6 months
- The AUDIT-C would be better as it is a quantity frequency measure that is widely used

2. b) Please explain your answer in 2 a).

- 12 months is more likely to be difficult for the participant to recall and likely to result in more reporting errors. Weekly intake might improve reporting accuracy.
- alc consumption over the past 12 months is the most representative and stable measure. Drinking in the past 7 days and beverage preferences are auxilliary traits that might lead to more detailed clusters being identified, but including these in the first instance risk obscuring the main patterns in clustering that you would want to drive your conclusions
- People are bad at reporting quantity and past 7 day frequency likely to be influenced by seasonal fluctuations.
- Frequency of drinking alone is insufficient to predict risk (someone binge-drinking on one day a week might be at greater risk than someone drinking 1 UK every day)
- This suggestion is based on the literature suggesting that a short recall period is preferable to a long one, particularly when asking participants about routine or frequent events
- Quantity in last 7 days will reflect frequency and will be much better recalled than frequency in last 12 months...
- I've chosen this for pragmatic reasons - it's in the survey. Ideally you'd want to know average normal consumption in units. The past 7 days is fairly reliable though you may have to adjust for seasonality etc. (people drink more at Christmas)
- see above
- Quantity over a period of time takes priority for me over frequency. My justification for this is that we have clear guidelines over recommended units. Most relevant to behavioural clustering (amount/units relevant to risk of harmful drinking)

2. c) Given the measure of alcohol consumption you chose in 2. a), how many categories would you choose?

- I would avoid categories (use continuously), or categorise based on the data collected (e.g. moderation analysis or hierarchial cluster analysis) abst, lower, med, high risk as usual by SARG
- Maybe 5 or so - but this may seem excessive compared to simple smoking categorisation 3
- 3: Lower risk; increasing risk; high risk

2. d) What cut-off points would you use to categorise alcohol consumption?

- 0, 1-14, 14-35 women, 14-50 men, >35 women, >50 men (UK standard units / week)
- 0, 1-14, 14-28, 28+ units or similar
- Daily / almost daily (including 5-6 per week), another category which basically incorporates the old CMO guidance on spreading drinking over 3 or more days, and then a final category which is basically abstainers last two categories)
- Cut-offs used in HSE: 0-13: lower risk; 14-35(women), 14-50(men): increasing risk; 36+(women) 51+(men): high risk number of drinks consumed i.e. 4 pints of beer (I'd then do the conversion to units yourself. Folk don't work in units)

FRUIT AND VEGETABLE INTAKE

The English Longitudinal Study of Ageing study collects information on fruit and vegetable intake on a typical day.

3. a) How would you categorise fruit and vegetable intake and what cut-offs would you use? (Note : Since ELSA has only one measure of nutrition, we are only interested in how this measure of nutrition can be categorised.) The WHO recommends consuming five portions of fruit and vegetables a day. Thus, one way to categorise participants' fruit and vegetable intake would be to divide it into two categories with five portions as the cut-off. i) Do you think it is appropriate to categorise participants into those who meet versus those who do not meet the WHO recommendation?

Yes	77%
No	23%

3. b) Based on the measures used in the ELSA study, how many categories would you divide participants' fruit and vegetable intake into?

- Similar to before, I would avoid categories. But if you need to have them, I would recommend having more than two to capture low and high f/v intake
- Intuitively meeting WHO recommendations makes perfect sense, my only concern is that the average intake of F&V is 2.6 portions in the UK and so you may find it doesn't differentiate groups. Perhaps consider a split based on the median in the population
- 0-1; 2-4; 5+
-
- 3. c) What cut-offs points would you use for each category?
-
- Similar to before, I would avoid categories. But if you need to have them, I would recommend having more than two to capture low and high f/v intake
- Intuitively meeting WHO recommendations makes perfect sense, my only concern is that the average intake of F&V is 2.6 portions in the UK and so you may find it doesn't differentiate groups. Perhaps consider a split based on the median in the population
- 0-1; 2-4; 5+

PHYSICAL ACTIVITY

For physical activity, the English Longitudinal Study of Ageing study collects data on how often (i.e. more than once a week, 2 once a week, 3 one to three times a month, 4 hardly ever, or never?) an individual engages in physical activity at the following intensities :

- **mild (laundry and home repairs)**
- **moderate (gardening, cleaning the car, walking at moderate pace, dancing)**
- **vigorous (e.g., running/ jogging, swimming, cycling, aerobics/gym workout, tennis, and digging with a spade)**

4. a) Based on the above stated measures, some studies have categorised physical activity as : - Sedentary: light exercise 1–3 times a month, no moderate or vigorous activity - Low: light exercise at least once a week but no vigorous activity - Moderate: moderate activity more than once a week, or vigorous activity between once a week to 1–3 times a month - High: vigorous activity more than once a week. i) Do you think this would be an appropriate way to categorise physical activity based on the measures used in the ELSA study? (Note : Since ELSA has only one measure of physical activity, we are only interested in how this measure of physical activity can be categorised.)

Yes	73%
No	27%

4. b) How would you categorise physical activity based on the above stated measures used in the ELSA study?

- I would avoid categories, but if needed, would set moderate as meeting the UK recommended PA guidelines. High would be those reporting physical activity levels above the recommendations.
- The evidence on physical activity in the latest guidance recognises the role that higher volumes of light intensity physical activity can play in health outcomes. This is in addition to moderate and vigorous physical activity. The categories suggested above prioritise vigorous activity and yet a person could engage in one bout of vigorous physical activity per week and be sedentary for the rest of the time. This would not relate to a category of 'highly active'. To overcome this, you could use a combination of mild, moderate and vigorous activity in your categorisation by applying a score to each of the frequencies (1-5) in each of the categories and then using these scores to create categories. In this instance the maximum score would be 15 and the minimum would be 0. Categories could then be 1) A total score of less then 3 = sedentary 2) A total score of 3 - 8 = low 3) A total score of 8 -12 is moderate and 12 plus = high.

Fig S1. Conversion rates for fruit and vegetable intake in Wave 4

35 Using the measures below, how much of the following did you eat yesterday?
Please read through the whole list before answering.
For each food type, write '0' if none eaten.

	Write in number	
Salad (cereal bowlfuls)	☐☐	x 1
Tablespoons of vegetables (raw, cooked, frozen or tinned) *Include peas and greens. Do not include potatoes*	☐☐	x 1/3
Tablespoons of pulses such as baked beans, red kidney beans, lentils	☐☐	x 1/3 (up to 1)
Tablespoons of other dishes mainly made from vegetables or pulses, such as vegetable lasagne or vegetable curry	☐☐	x 1/3

36 Using the measures below, how much of the following did you eat yesterday?
Please read through the whole list before answering.
For each food type, write '0' if none eaten.

	Write in number	
Average handfuls of very small fruit, such as grapes, berries	☐☐	x 1/2
Small fruit, such as plums, satsumas	☐☐	x 1/2
Medium fruit, such as apples, bananas, oranges	☐☐	x 1
Half a large fruit, such as grapefruit	☐☐	x 1
Average slices of a very large fruit, such as melon	☐☐	x 1
Tablespoons of frozen or tinned fruit	☐☐	x 1/3
Tablespoons of dried fruit, such as raisins, apricots	☐☐	x 1 (up to 1)
Tablespoons of other dishes made mainly from fruit such as fruit salad or fruit pies	☐☐	x 1/3
Small glasses of fruit juice	☐☐	x 1 (up to 1)

Note. "The amount of consumed fruit and vegetables were converted into portions (1 portion = 80 g for both vegetables and fruit) in accordance with the Welsh Health Survey methodology and the "5 A Day" campaign portion size from the National Health Service (NHS)."[1]

Section 2: Supplementary Missing Data Analysis

As seen in Table S2, the included sample had a slightly higher average age (62.9 years vs 60.4, p<0.001), a higher proportion of individuals in intermediate level jobs (26.2% vs. 21.6%, p=0.006) and professional/managerial occupations (39.3% vs. 30.1%, p<0.001). The included sample also had a higher proportion of individuals educated up to degree level or higher (20.9% vs 16.7%, p=0.015) and a higher proportion of individuals with parents in intermediate occupations (32.1% vs. 25.8%, p=0.006) than the excluded sample. There were no significant differences in sex, other socio-demographic variables, or disease status between the included and excluded samples, except for complex multimorbidity, respiratory disorders and endocrine disorders (see Table S2). In sum, the absolute differences between the included and excluded samples were not substantial, though they achieved significance in some cases due to the relatively large number of participants.

Table S2. Comparison of the included sample (i.e., with no missing data on any sociodemographic variables, n=4759) and the excluded sample (i.e., with missing on at least one sociodemographic variable, n=670)

Baseline socio-demographic characteristics	Included sample (n=4759) N (%)	Excluded sample (n=670) N (%)	Test estimate	p-value
Male	2081 (43.7)	271 (40.4)	2.44	0.118
Female	2678 (56.3)	399 (59.6)	2.44	0.118
Average Age (s.d.)	62.9 (8.1)	60.4 (8.2)	**7.65***	**<0.001***
Parental Occupation – Semi-routine and routine	1299 (27.3)	163 (34.8)	**11.42***	**<0.001***
Parental Occupation – Intermediate	1530 (32.1)	121 (25.8)	**7.68***	**0.006***
Parental Occupation – Professional/managerial	1930 (40.6)	185 (39.4)	0.17	0.676
Occupation – Semi-routine and routine	1646 (34.6)	221 (48.3)	**33.36***	**<0.001***
Occupation – Intermediate	1245 (26.2)	99 (21.6)	**4.28***	**0.039***
Occupation – Professional/managerial	1868 (39.3)	138 (30.1)	**14.30***	**<0.001***
Education – No qualifications	958 (20.1)	172 (26.8)	**14.77***	**<0.001***
Education – Intermediate	2807 (59)	363 (56.5)	1.29	0.256
Education – Degree/higher	994 (20.9)	107 (16.7)	**5.95***	**0.015***
Wealth – First Tertile	1260 (26.5)	127 (33.1)	**7.70***	**0.006***
Wealth – Second Tertile	1615 (33.9)	119 (31.1)	1.19	0.278
Wealth – Third Tertile	1884 (39.6)	137 (35.8)	2.01	0.156
**Disease status at Wave 4**				
Multimorbidity	1802 (37.9)	263 (39.3)	0.42	0.515
Complex multimorbidity	588 (12.4)	113 (16.9)	**10.23***	**0.001***
Respiratory disorders	548 (11.5)	102 (15.2)	**7.38***	**0.006***
Eye disorders	729 (15.3)	93 (13.9)	0.82	0.365
Musculoskeletal and connective system disorders	1599 (33.6)	222 (33.2)	0.03	0.874
Neoplasms	140 (2.9)	18 (2.7)	0.06	0.814
Circulatory disorders	1870 (39.3)	259 (38.7)	0.06	0.806
Endocrine nutritional & metabolic disorders	334 (7)	68 (10.2)	**7.99**	**0.005***
**Disease status at Wave 9**				
Multimorbidity	2755 (57.9)	388 (57.9)	0	1.00
Complex multimorbidity	1278 (26.9)	208 (31)	**4.98***	**0.026***
Respiratory disorders	664 (14)	115 (17.2)	**4.80***	**0.028***
Eye disorders	1879 (39.5)	253 (37.9)	0.60	0.441
Musculoskeletal and connective system disorders	2261 (47.5)	323 (48.4)	0.14	0.713
Neoplasms	235 (4.9)	27 (4)	0.84	0.359
Circulatory disorders	2394 (50.3)	350 (52.3)	0.87	0.351
Endocrine nutritional & metabolic disorders	621 (13)	117 (17.5)	**9.51***	**0.002***

Note. The differences for all variables except age were calculated using the two-sample test for equality of proportions. For age, the two-sample Welsh t-test was used to evaluate the differences. ***p<0.05**

Section 3: Supplementary Statistical Analysis

For the likelihood-based tests such as the VLMR-LMR test, a p-value < 0.05 indicates that the model fit has not significantly improved compared to the model with one less class. Lower values of the BIC, CAIC, saBIC, and AWE indicate a better fitting model. However, when assessing the best model fit, particularly in large datasets with multiple indicators, additional classes can often lead to a decrease in the information criterion (ICs) — favouring the more complex model — until no further class can be added due to convergence issues.[2] Thus, there exists no global minimum. In such cases, the recommendation is to plot the ICs to seek a point of inflection or plateauing.[2] Finally, entropy and the smallest average latent class posterior probability were also used to quantify how well the model separated individuals into distinct latent classes, with values closer to 1 indicating distinct separation between classes.

Table S3. Disease status of the participants (N= 4759) at Wave 9 (2018-2019)

**Disease status at Wave 9 (2018-2019)**		
Multimorbidity, N(%)	Has condition	2755 (57.9)
	Missing	..
Complex multimorbidity, N(%)	Has condition	1278 (26.9)
	Missing	..
Respiratory disorders, N(%)	Has condition	664 (14)
	Missing	1 (<0.1)
Eye disorders, N(%)	Has condition	1879 (39.5)
	Missing	4 (0.1)
Musculoskeletal and connective system disorders, N(%)	Has condition	2261 (47.5)
	Missing	..
Neoplasms, N(%)	Has condition	235 (4.9)
	Missing	1 (0)
Circulatory disorders, N(%)	Has condition	2394 (50.3)
	Missing	..
Endocrine nutritional & metabolic disorders, N(%)	Has condition	621 (13)
	Missing	4 (0.1)
Nervous system disorders, N(%)	Has condition	168 (3.5)
	Missing	4530 (95.2) *

Mental & behavioural issues, N(%)	Has condition	63 (1.3)
	Missing	4688 (98.5) *

* Due to a large proportion of missing data on disease status for two health conditions i.e., nervous system disorders (95.2%), and mental and behavioural issues (98.5%), they were excluded from subsequent analyses.

Fig S2. Elbow plot of Bayesian Information Criteria and other indices for model fitting

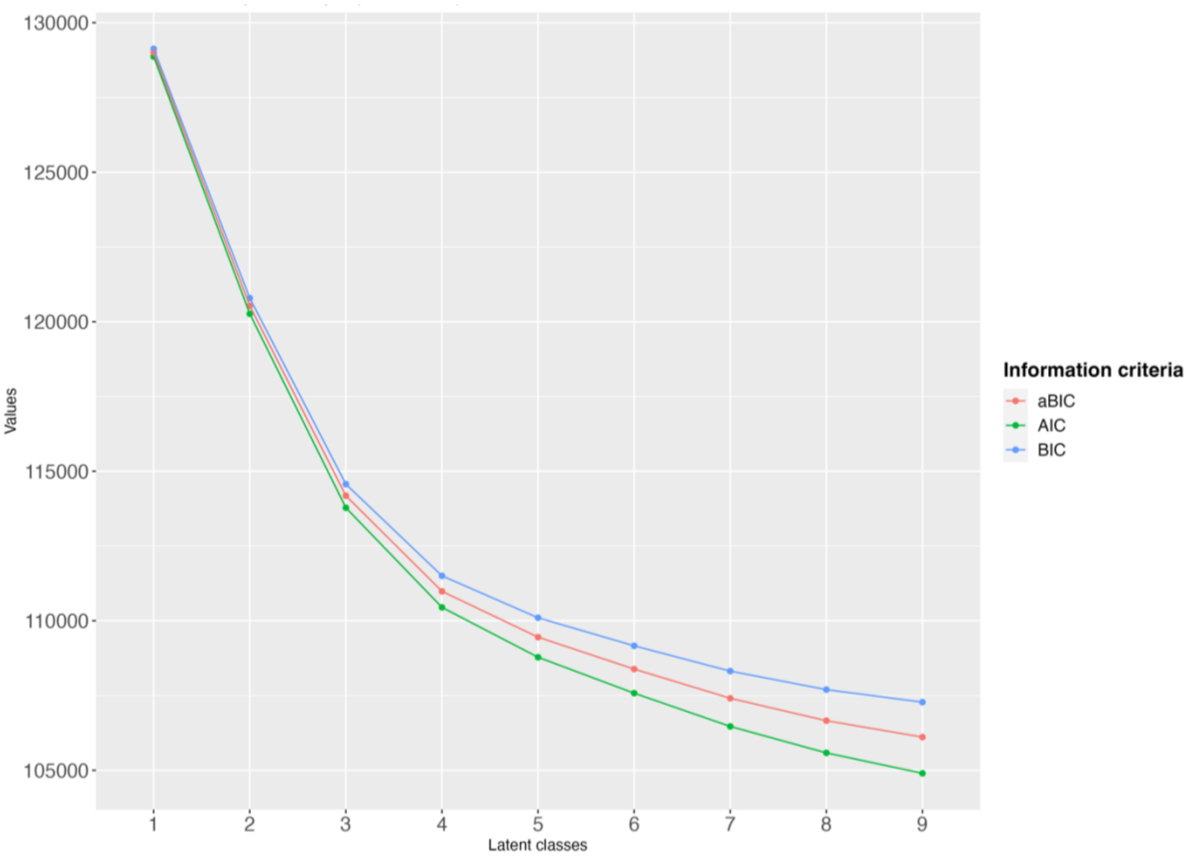

Note. BIC = Bayesian information criterion; SABIC = sample size adjusted BIC; CAIC = consistent Akaike information criterion

Section 4: Split-half replication

To ensure the reliability of our class solution, the sample (n=4759) was randomly split into halves and separate, sequential latent class models were conducted on both random subsamples. These subsamples were named Sample A (n=2379) and Sample B (n=2380). We then compared the model fit statistics (such as the Bayesian information criterion, sample size adjusted BIC, consistent Akaike information criterion, and loglikelihoods) of the three samples using screeplots. If the final latent class solution (i.e., a seven-class model) was consistently identified as the best fit in all three samples, then this would provide evidence for the replicability of the latent classes identified in the analysis. Fig S3 shows the screeplots for the model fit statistics in the three samples. Screeplots of model fit statistics for one to nine class solutions in the random subsamples and those for the whole sample are shown in Fig S3. In all three samples, the decline in the information criteria levelled off around seven classes, providing initial evidence for replication of the results across the three samples. Additionally, we compared the structures (i.e., the prevalence of latent classes and the probabilities of item responses) of the seven-class model with the five-class and six-class models in the three samples (see Supplementary Figures 4-6). We found that the seven-class model replicated with maximum stability (i.e., had a similar latent class structure) across all three samples. On the other hand, the five-class and six-models were unstable as they did not replicate across all the three samples.

Fig S3. Screeplots of Bayesian information criterion (BIC), Sample size adjusted BIC (SABIC); Consistent Akaike information criterion (CAIC) and loglikelihoods for i) Sample A (n=2379, 50% random split sample), ii) Sample B (n=2380, 50% random split sample) and iii) Complete Sample (n=4759).

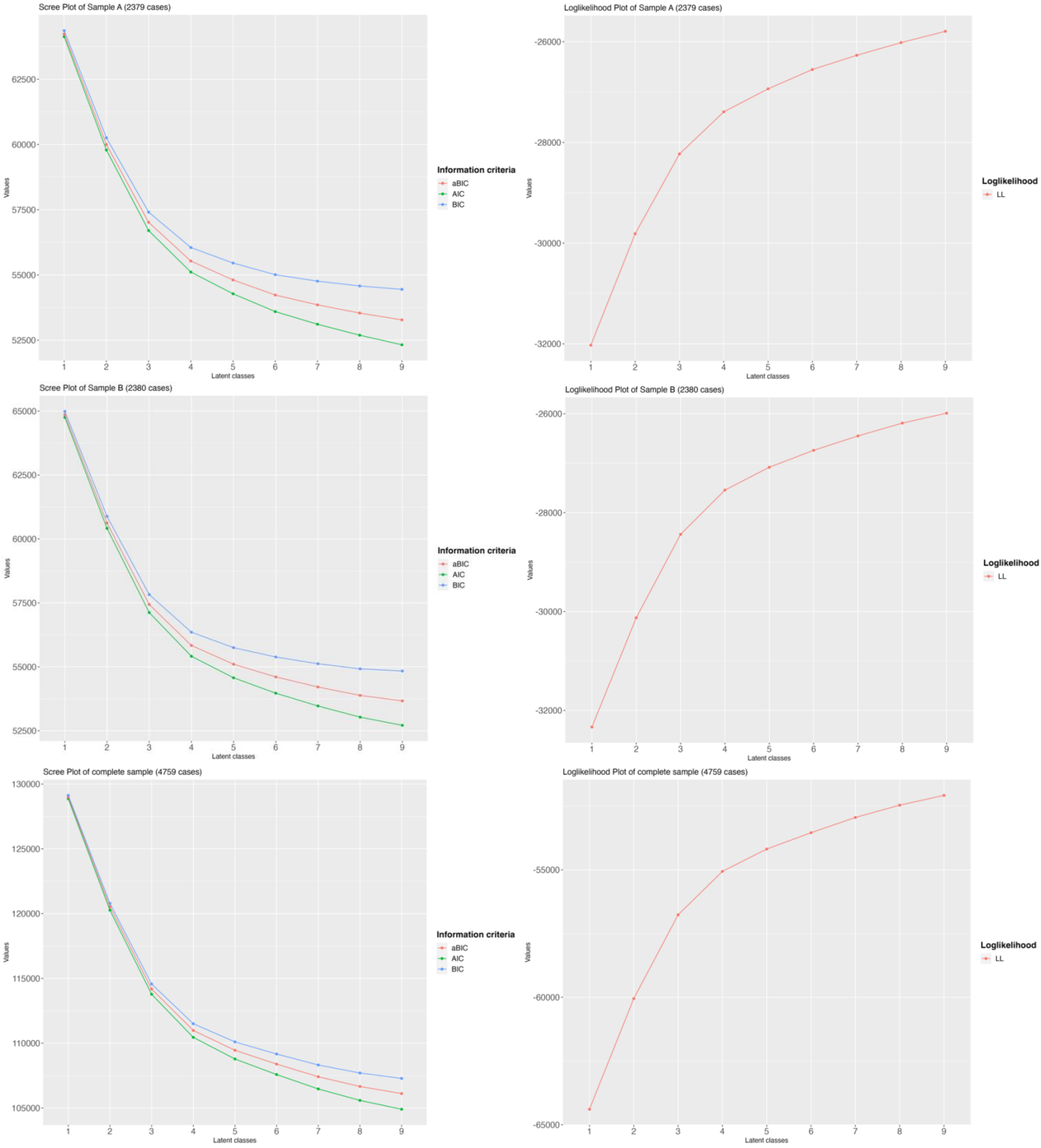

Fig S4. 5-class model solutions for Sample A (n=2379, 50% random split sample), Sample B (n=2380, 50% random split sample) and Complete Sample (n=4759)

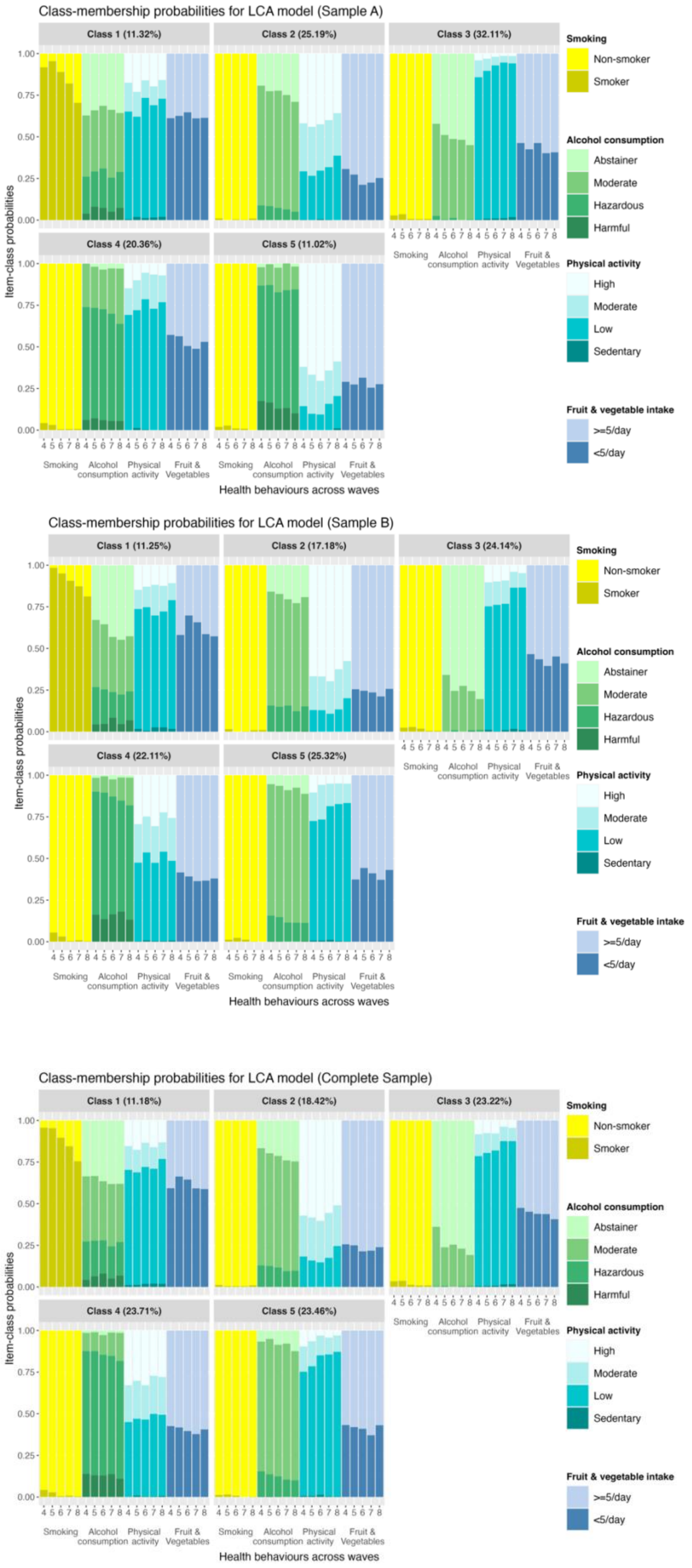

Fig S5. 6-class model solutions for Sample A (n=2379, 50% random split sample), Sample B (n=2380, 50% random split sample) and Complete Sample (n=4759)

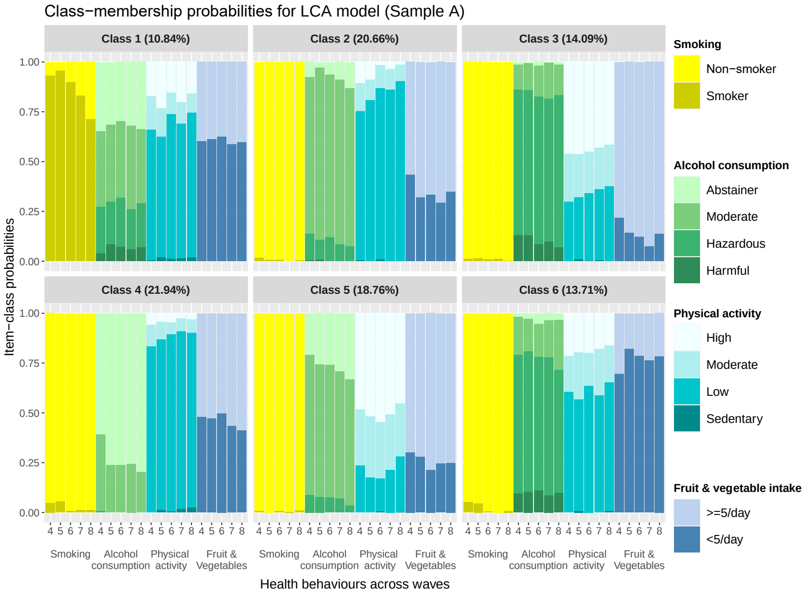

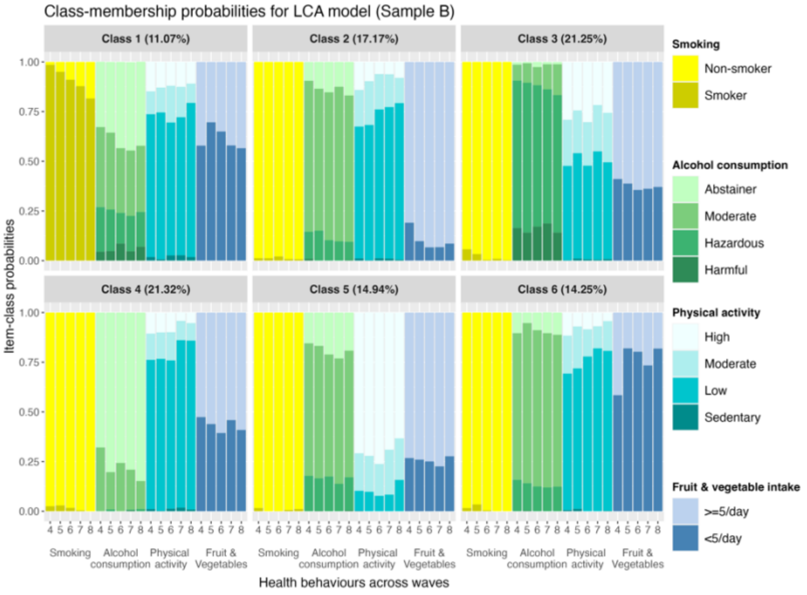

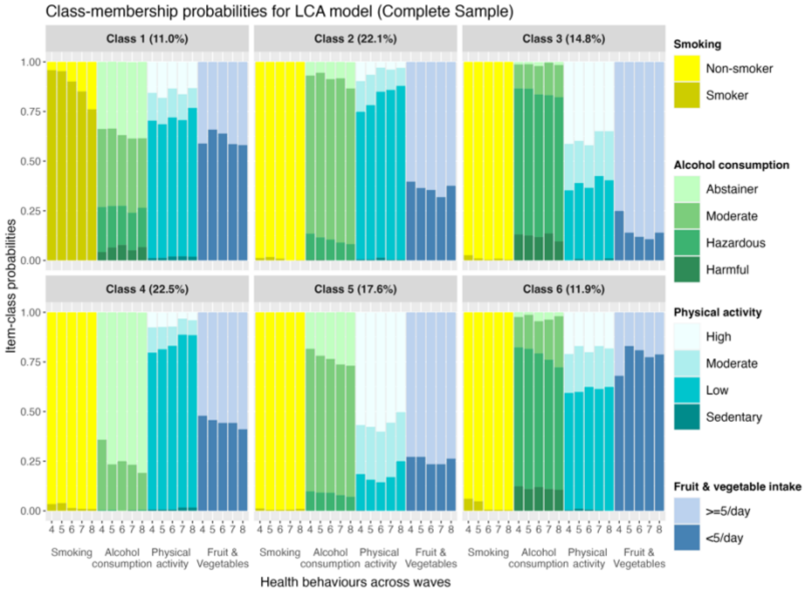

Fig S6. 7-class model solutions for Sample A (n=2379, 50% random split sample), Sample B (n=2380, 50% random split sample) and Complete Sample (n=4759)

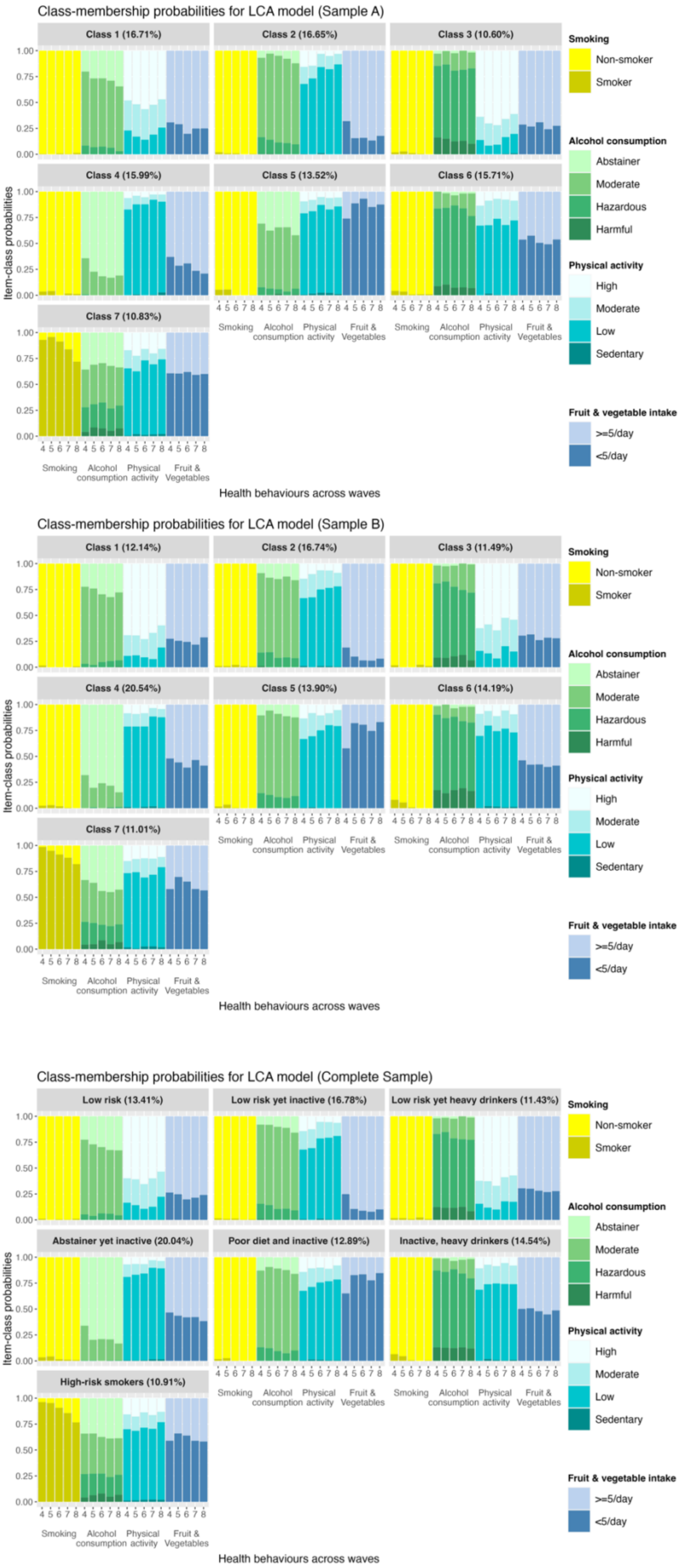

Table S5. Pairwise comparison of differences in disease status across latent classes (unadjusted for sociodemographic characteristics and baseline disease status)

Health conditions	Latent classes	Prevalence	1<br>Low risk (Ref. class)	2<br>Low risk yet inactive	3<br>Low risk yet heavy drinkers	4<br>Abstainers but inactive	5<br>Poor diet and inactive	6<br>Inactive, heavy drinkers	7<br>High-risk smokers
			(n=13.4 %)	(n=16.8%)	(n=11.4%)	(n=20 %)	(n=12.9 %)	(n=14.5 %)	(n=10.9 %)
**Multimorbidity**	1	**0.444**		**-0.212**	0.015	**-0.294**	**-0.154**	**-0.15**	**0.129**
				**(0.000)**	(0.692)	**(0.000)**	**(0.000)**	**(0.000)**	**(0.000)**
χ2 (6) = 123.432 , p= 0.000									
	2	**0.656**	**0.212**		**0.227**	-0.082	0.058	0.062	-0.083
			**(0.000)**		**(0.000)**	(0.012)	(0.124)	(0.070)	(0.019)
	3	**0.429**	-0.015	**-0.227**		**-0.309**	**-0.169**	**-0.165**	**0.144**
			(0.692)	**(0.000)**		**(0.000)**	**(0.000)**	**(0.000)**	**(0.000)**
	4	**0.738**	**0.294**	0.082	**0.309**		**0.14**	**0.144**	**-0.165**
			**(0.000)**	(0.012)	**(0.000)**		**(0.000)**	**(0.000)**	**(0.000)**
	5	**0.598**	**0.154**	-0.058	**0.169**	**-0.14**		0.004	-0.025
			**(0.000)**	(0.124)	**(0.000)**	**(0.000)**		(0.907)	(0.523)
	6	**0.594**	**0.15**	-0.062	**0.165**	**-0.144**	-0.004		-0.021
			**(0.000)**	(0.070)	**(0.000)**	**(0.000)**	(0.907)		(0.567)
	7	0.573	**0.129**	-0.083	**0.144**	**-0.165**	-0.025	-0.021	
			**(0.000)**	(0.019)	**(0.000)**	**(0.000)**	(0.523)	(0.567)	
**Complex Multimorbidity**	1	**0.16**		**-0.174**<br>**(0.000)**	0.051<br>(0.048)	**-0.269**<br>**(0.000)**	**-0.109**<br>**(0.001)**	**-0.099**<br>**(0.000)**	**0.124**<br>**(0.000)**
	2	**0.334**	**0.174**		**0.225**	**-0.095**	0.065	0.075	-0.05
χ2 (6) = 141.267 , p=0.000			**(0.000)**		**(0.000)**	**(0.004)**	(0.074)	(0.018)	(0.130)
	3	**0.109**	-0.051	**-0.225**		**-0.32**	**-0.16**	**-0.15**	**0.175**

			(0.048)	**(0.000)**		**(0.000)**	**(0.000)**	**(0.000)**	**(0.000)**
	4	**0.429**	**0.269**	**0.095**	**0.32**		**0.16**	**0.17**	**-0.145**
			**(0.000)**	**(0.004)**	**(0.000)**		**(0.000)**	**(0.000)**	**(0.000)**
	5	**0.269**	**0.109**	-0.065	**0.16**	**-0.16**		0.01	0.015
			**(0.001)**	(0.074)	**(0.000)**	**(0.000)**		(0.777)	(0.677)
	6	**0.259**	**0.099**	-0.075	**0.15**	**-0.17**	-0.01		0.025
			**(0.000)**	(0.018)	**(0.000)**	**(0.000)**	(0.777)		(0.437)
	7	0.284	**0.124**	-0.05	**0.175**	**-0.145**	0.015	0.025	
			**(0.000)**	(0.130)	**(0.000)**	**(0.000)**	(0.677)	(0.437)	
**Respiratory disorders**	1	**0.09**		-0.059	-0.003	**-0.097**	-0.016	-0.04	**0.137**
				(0.012)	(0.887)	**(0.000)**	(0.505)	(0.072)	**(0.000)**
χ2 (6) = 48.588 , p= 0.0000									
	2	**0.149**	0.059		0.056	-0.038	0.043	0.019	**0.078**
			(0.012)		(0.015)	(0.131)	(0.104)	(0.406)	**(0.004)**
	3	**0.093**	0.003	-0.056		**-0.094**	-0.013	-0.037	**0.134**
			(0.887)	(0.015)		**(0.000)**	(0.589)	(0.128)	**(0.000)**
	4	**0.187**	**0.097**	0.038	**0.094**		**0.081**	0.057	0.04
			**(0.000)**	(0.131)	**(0.000)**		**(0.005)**	(0.018)	(0.164)
	5	**0.106**	0.016	-0.043	0.013	**-0.081**		-0.024	**0.121**
			(0.505)	(0.104)	(0.589)	**(0.005)**		(0.363)	**(0.000)**
	6	**0.13**	0.04	-0.019	0.037	-0.057	0.024		**0.097**
			(0.072)	(0.406)	(0.128)	(0.018)	(0.363)		**(0.000)**
	7	**0.227**	**0.137**	**0.078**	**0.134**	0.04	**0.121**	**0.097**	
			**(0.000)**	**(0.004)**	**(0.000)**	(0.164)	**(0.000)**	**(0.000)**	
**Endocrine,**	1	**0.074**		**-0.083**	0.027	**-0.173**	**-0.074**	-0.026	**0.065**
**nutritional and**				**(0.001)**	(0.162)	**(0.000)**	**(0.004)**	(0.181)	**(0.005)**

**metabolic**	2	**0.157**	**0.083**		**0.11**	**-0.09**	0.009	0.057	-0.018
**disorders**			**(0.001)**		**(0.000)**	**(0.001)**	(0.764)	(0.019)	(0.500)
$\chi2$ (6) = 90.007 , p= 0.000	3	**0.047**	-0.027	**-0.11**		**-0.2**	**-0.101**	-0.053	**0.092**
			(0.162)	**(0.000)**		**(0.000)**	**(0.000)**	(0.014)	**(0.000)**
	4	**0.247**	**0.173**	**0.09**	**0.2**		**0.099**	**0.147**	**-0.108**
			**(0.000)**	**(0.001)**	**(0.000)**		**(0.003)**	**(0.000)**	**(0.000)**
	5	**0.148**	**0.074**	-0.009	**0.101**	**-0.099**		0.048	-0.009
			**(0.004)**	(0.764)	**(0.000)**	**(0.003)**		(0.075)	(0.763)
	6	**0.1**	0.026	-0.057	0.053	**-0.147**	-0.048		0.039
			(0.181)	(0.019)	(0.014)	**(0.000)**	(0.075)		(0.110)
	7	0.139	**0.065**	-0.018	**0.092**	**-0.108**	-0.009	0.039	
			**(0.005)**	(0.500)	**(0.000)**	**(0.000)**	(0.763)	(0.110)	
**Circulatory disorders**	1	**0.411**		**-0.125**	0.015	**-0.221**	-0.098	**-0.127**	**0.116**
$\chi2$ (6) = 66.513 , p= 0.000				**(0.001)**	(0.683)	**(0.000)**	(0.012)	**(0.000)**	**(0.002)**
	2	**0.536**	**0.125**		**0.14**	**-0.096**	0.027	-0.002	-0.009
			**(0.001)**		**(0.000)**	**(0.005)**	(0.485)	(0.947)	(0.812)
	3	**0.396**	-0.015	**-0.14**		**-0.236**	**-0.113**	**-0.142**	**0.131**
			(0.683)	**(0.000)**		**(0.000)**	**(0.004)**	**(0.000)**	**(0.000)**
	4	**0.632**	**0.221**	**0.096**	**0.236**		**0.123**	**0.094**	**-0.105**
			**(0.000)**	**(0.005)**	**(0.000)**		**(0.001)**	**(0.005)**	**(0.003)**
	5	**0.509**	0.098	-0.027	**0.113**	**-0.123**		-0.029	0.018
			(0.012)	(0.485)	**(0.004)**	**(0.001)**		(0.450)	(0.644)
	6	**0.538**	**0.127**	0.002	**0.142**	**-0.094**	0.029		-0.011
			**(0.000)**	(0.947)	**(0.000)**	**(0.005)**	(0.450)		(0.765)
	7	0.527	**0.116**	-0.009	**0.131**	**-0.105**	0.018	-0.011	

			**(0.002)**	(0.812)	**(0.000)**	**(0.003)**	(0.644)	(0.765)	
**Eye disorders**	1	**0.323**		**-0.129**	0.012	**-0.163**	**-0.113**	-0.056	-0.01
$\chi 2$ (6) = 57.967 , p= 0.000				**(0.000)**	(0.722)	**(0.000)**	**(0.003)**	(0.087)	(0.751)
	2	**0.452**	**0.129**		**0.141**	-0.034	0.016	0.073	**-0.139**
			**(0.000)**		**(0.000)**	(0.326)	(0.677)	(0.034)	**(0.000)**
	3	**0.311**	-0.012	**-0.141**		**-0.175**	**-0.125**	-0.068	0.002
			(0.722)	**(0.000)**		**(0.000)**	**(0.001)**	(0.057)	(0.960)
	4	**0.486**	**0.163**	0.034	**0.175**		0.05	**0.107**	**-0.173**
			**(0.000)**	(0.326)	**(0.000)**		(0.196)	**(0.001)**	**(0.000)**
	5	**0.436**	**0.113**	-0.016	**0.125**	-0.05		0.057	**-0.123**
			**(0.003)**	(0.677)	**(0.001)**	(0.196)		(0.135)	**(0.001)**
	6	**0.379**	0.056	-0.073	0.068	**-0.107**	-0.057		-0.066
			(0.087)	(0.034)	(0.057)	**(0.001)**	(0.135)		(0.051)
	7	0.313	-0.01	**-0.139**	0.002	**-0.173**	**-0.123**	-0.066	
			(0.751)	**(0.000)**	(0.960)	**(0.000)**	**(0.001)**	(0.051)	
**Musculoskeletal disorders**	1	**0.392**		-0.152	0.01	-0.191	-0.075	-0.058	0.106
$\chi 2$ (6) = 58.410 , p= 0.000				(0.544)	(0.256)	(0.254)	(0.146)	(0.402)	(0.607)
	2	**0.544**	0.152		0.162	-0.039	0.077	0.094	-0.046
			(0.544)		(0.540)	(0.556)	(0.334)	(0.823)	(0.947)
	3	**0.382**	-0.01	-0.162		-0.201	-0.085	-0.068	0.116
			(0.256)	(0.540)		(0.957)	(0.725)	(0.701)	(0.529)
	4	**0.583**	0.191	0.039	0.201		0.116	0.133	-0.085
			(0.254)	(0.556)	(0.957)		(0.682)	(0.712)	(0.540)
	5	**0.467**	0.075	-0.077	0.085	-0.116		0.017	0.031
			(0.146)	(0.334)	(0.725)	(0.682)		(0.457)	(0.349)

6	**0.45**	0.058	-0.094	0.068	-0.133	-0.017		0.048
		(0.402)	(0.823)	(0.701)	(0.712)	(0.457)		(0.789)
7	0.498	0.106	-0.046	0.116	-0.085	0.031	0.048	
		(0.607)	(0.947)	(0.529)	(0.540)	(0.349)	(0.789)	

Note. The omnibus Wald test results were significant for all eight disease outcomes was significant except Neoplasm $\chi 2$ (6) = 7.263, p= 0.297. Significant omnibus Wald test results indicate that the prevalence of these health outcomes differed across clusters and were followed up with pairwise Wald tests. The estimates shown above are the absolute differences in proportions of participants having a given health outcome in the class (in row) minus the class (in column). **Bold values are statistically significant at the Bonferroni-corrected significance level (p=0.007)** and indicate the two-tailed p-values for pairwise Wald test for differences in proportion for the class (in row) minus the class (in column).

Section 5: STROBE Statement—checklist of items that should be included in reports of observational studies

	Item No.	Recommendation	Page No.	Relevant text from manuscript
**Title and abstract**	1	(*a*) Indicate the study's design with a commonly used term in the title or the abstract	Abstract, second paragraph	"Longitudinal study"
		(*b*) Provide in the abstract an informative and balanced summary of what was done and what was found	Abstract, paragraph 2-3	
**Introduction**				
Background/rationale	2	Explain the scientific background and rationale for the investigation being reported	Introduction, paragraphs 1-5	
Objectives	3	State specific objectives, including any prespecified hypotheses	Introduction, paragraph 6	"Our research analyses data from a longitudinal panel of older adults in England to: i) explore how the SNAP behaviours cluster over time in older adults, ii) investigate how membership in different behavioural clusters varies by socio-demographic characteristics, and iii) examine which, if any, behavioural clusters are prospectively associated with multimorbidity over time. "
**Methods**				
Study design	4	Present key elements of study design early in the paper	Methods, paragraph 1	
Setting	5	Describe the setting, locations, and relevant dates, including periods of recruitment, exposure, follow-up, and data collection	Methods, paragraph 1	"We analysed secondary data from the English Longitudinal Study of Ageing (ELSA) – a nationally representative, ongoing panel study of community-dwelling adults aged 50 and over at baseline, in England [27]. ELSA collects biennial data on mental and physical health, finances, and

				attitudes around ageing using computer-assisted interviews and questionnaires [27].”
Participants	6	(*a*) *Cohort study*—Give the eligibility criteria, and the sources and methods of selection of participants. Describe methods of follow-up<br>*Case-control study*—Give the eligibility criteria, and the sources and methods of case ascertainment and control selection. Give the rationale for the choice of cases and controls<br>*Cross-sectional study*—Give the eligibility criteria, and the sources and methods of selection of participants	Methods, paragraph 2-3	“Our analysis used data from 5,429 respondents to the core questionnaire across six waves from Wave 4 (2008-2009) to Wave 9 (2018-2019). We applied the longitudinal weights that were provided with the dataset and had been derived using information spanning from Wave 4 to Wave 9 to reduce drop-out bias. Wave 4 was selected as the baseline because, although data on health behaviours was available from Wave 3, longitudinal weights were only available from Wave 1 or Wave 4. Choosing Wave 3 as the baseline would have resulted in the loss of data on approximately 2000 participants due to longitudinal weighting.<br><br>Participants (n=670) with missing values on socio-demographic variables were removed using listwise deletion, leaving a final sample of 4759 participants (87.6% of the original sample; see Fig 1).”
		(*b*) *Cohort study*—For matched studies, give matching criteria and number of exposed and unexposed<br>*Case-control study*—For matched studies, give matching criteria and the number of controls per case	N/a	
Variables	7	Clearly define all outcomes, exposures, predictors, potential confounders, and effect modifiers. Give diagnostic criteria, if applicable	Methods, paragraph 5-13	

Data sources/ measurement	8	For each variable of interest, give sources of data and details of methods of assessment (measurement). Describe comparability of assessment methods if there is more than one group	Methods, paragraph 5-13	
Bias	9	Describe any efforts to address potential sources of bias	Methods, paragraph 4, Supplementary Section 2	"To assess the potential impact of excluding participants with missing data, we compared the included and excluded samples (for details, see Supplementary Section 2). Overall, the absolute differences between the included and excluded samples were not substantial, though differences for some socio-demographic variables (average age, tertiary education, intermediate and professional/managerial occupations) and disease status (complex multimorbidity, respiratory disorders and endocrine disorders) achieved significance due to the relatively large number of participants."
Study size	10	Explain how the study size was arrived at	Methods, paragraph 2-3, Figure 1	"Our analysis used data from 5,429 respondents to the core questionnaire across six waves from Wave 4 (2008-2009) to Wave 9 (2018-2019). We applied longitudinal weights from Wave 4 to reduce drop-out bias. Wave 4 was selected as the baseline because although health behaviour data were available starting in Wave 3, longitudinal weights were only available for Waves 1 and 4. Choosing Wave 3 as the baseline would have resulted in the loss of data on

	approximately 2000 participants due to longitudinal weighting. Participants (n=670) with missing values on one or more socio-demographic variables were removed using listwise deletion, leaving a final effective sample of 4759 participants (87.6% of the original sample)”

Continued on next page

Quantitative variables	11	Explain how quantitative variables were handled in the analyses. If applicable, describe which groupings were chosen and why	Methods, paragraph 5-13	
Statistical methods	12	(*a*) Describe all statistical methods, including those used to control for confounding	Methods, paragraph 14-17	" RMLCA was used to examine whether there were distinct classes of respondents who had similar patterns of SNAP behaviours over time. RMLCA was chosen as it adopts a probabilistic model-based approach for capturing the number and composition of clusters, handles categorical data well, and allows for reliable interpretation and replication of patterns uncovered in the data. MPlus v8.5 software and R version v4.0.3 [41, 42] was used to conduct the RMLCA. A two-stage approach was used...."
		(*b*) Describe any methods used to examine subgroups and interactions	N/A	
		(*c*) Explain how missing data were addressed	Methods, paragraph 3	" Participants (n=670) with missing values on socio-demographic variables were removed using listwise deletion, leaving a final sample of 4759 participants (87.6% of the original sample; see Fig 1). We chose to use listwise deletion because the MPlus v8.5 software package does not support handling missing data for socio-demographic predictors in a latent class analysis (see Figure 1). More specifically, data was missing for occupation (n=212), education (n=28), wealth (n=287), and parental occupation (n=201). Given that no socio-demographic variable had more than 5% missing data, a threshold below which multiple imputation is

				deemed less beneficial, we favoured a complete case analysis [29]."
		(*d*) *Cohort study*—If applicable, explain how loss to follow-up was addressed<br>*Case-control study*—If applicable, explain how matching of cases and controls was addressed<br>*Cross-sectional study*—If applicable, describe analytical methods taking account of sampling strategy	N/a	
		(*e*) Describe any sensitivity analyses	Methods paragraph 15, Supplementary Section 4	"To assess the reliability of the class solution, we conducted a split-half replication where the sample was randomly split in half, and the above RMLCA was performed separately on these split samples to see if the solution for the full sample was replicated between these smaller splits."
**Results**				
Participants	13	(a) Report numbers of individuals at each stage of study—eg numbers potentially eligible, examined for eligibility, confirmed eligible, included in the study, completing follow-up, and analysed	N/a	
		(b) Give reasons for non-participation at each stage	N/a	
		(c) Consider use of a flow diagram	Figure 1	
Descriptive data	14*	(a) Give characteristics of study participants (eg demographic, clinical, social) and information on exposures and potential confounders	Results paragraph 1, Table 2	"Participants had an average age of 62.9 years (SD = 8.1) and approximately half were female (56.3%; see Table 2 for baseline demographic data). The sample's engagement in health behaviours across waves is shown in Table 3. The body system disorders with the highest prevalence were: multimorbidity (57.9%), circulatory disorders (50.3%), and disorders of the musculoskeletal and connective system (47.5%) (see Supplementary Table 3)."
		(b) Indicate number of participants with missing data for each variable of interest	Supplementary Table 2	

		(c) *Cohort study*—Summarise follow-up time (eg, average and total amount)	N/a
Outcome data	15*	*Cohort study*—Report numbers of outcome events or summary measures over time	Supplementary Table 2
		Case-control study—Report numbers in each exposure category, or summary measures of exposure	N/a
		Cross-sectional study—Report numbers of outcome events or summary measures	N/a
Main results	16	(*a*) Give unadjusted estimates and, if applicable, confounder-adjusted estimates and their precision (eg, 95% confidence interval). Make clear which confounders were adjusted for and why they were included	Table 5, Table 6, Supplementary Table 5
		(*b*) Report category boundaries when continuous variables were categorized	N/a
		(*c*) If relevant, consider translating estimates of relative risk into absolute risk for a meaningful time period	N/a

Continued on next page

Other analyses	17	Report other analyses done—eg analyses of subgroups and interactions, and sensitivity analyses	Supplementary Section 4, Supplementary figures 3-6	
**Discussion**				
Key results	18	Summarise key results with reference to study objectives	Discussion, paragraph 1-4	
Limitations	19	Discuss limitations of the study, taking into account sources of potential bias or imprecision. Discuss both direction and magnitude of any potential bias	Discussion, paragraph 6	
Interpretation	20	Give a cautious overall interpretation of results considering objectives, limitations, multiplicity of analyses, results from similar studies, and other relevant evidence	Discussion paragraphs 2-4, 8	
Generalisability	21	Discuss the generalisability (external validity) of the study results	Discussion, paragraph 6	"Finally, missing data are unavoidable in general population cohorts such as ELSA. We excluded participants with missing sociodemographic data at baseline. Those who were included were slightly older, were better educated, and were more likely to have more intermediate and professional level jobs than those who were excluded, meaning selection bias due to non-random exclusion is possible. This may limit the generalisability of our findings. Finally, as there are few ethnic minority participants in ELSA, our findings may not generalise to non-white populations."
**Other information**				
Funding	22	Give the source of funding and the role of the funders for the present study and, if applicable, for the original study on which the present article is based	Funding section	

References

1. Kojima G, Iliffe S, Jivraj S, Walters K. Fruit and Vegetable Consumption and Incident Prefrailty and Frailty in Community-Dwelling Older People: The English Longitudinal Study of Ageing. Nutrients. 2020;12(12):3882.

2. Nylund-Gibson K, Choi AY. Ten frequently asked questions about latent class analysis. Translational Issues in Psychological Science. 2018;4(4):440.
